# Functional Analysis of Human and Feline Coronavirus Cross-Reactive Antibodies Directed Against the SARS-CoV-2 Fusion Peptide

**DOI:** 10.3389/fimmu.2021.790415

**Published:** 2022-01-05

**Authors:** Nathalie Vanderheijden, Annelies Stevaert, Jiexiong Xie, Xiaolei Ren, Cyril Barbezange, Sam Noppen, Isabelle Desombere, Bruno Verhasselt, Peter Geldhof, Nick Vereecke, Veerle Stroobants, Dayoung Oh, Merijn Vanhee, Lieve M. J. Naesens, Hans J. Nauwynck

**Affiliations:** ^1^ Laboratory of Virology, Department of Translational Physiology, Infectiology and Public Health, Faculty of Veterinary Medicine, Ghent University, Merelbeke, Belgium; ^2^ Rega Institute for Medical Research, Department of Microbiology, Immunology and Transplantation, KU Leuven – University of Leuven, Leuven, Belgium; ^3^ National Influenza Centre and Epidemiology of Infectious Diseases, Sciensano, Brussels, Belgium; ^4^ Immune Response, Sciensano, Brussels, Belgium; ^5^ Laboratory for Medical Microbiology, Ghent University Hospital, Ghent, Belgium; ^6^ Laboratory of Parasitology, Department of Translational Physiology, Infectiology and Public Health, Faculty of Veterinary Medicine, Ghent University, Merelbeke, Belgium; ^7^ PathoSense BV, Lier, Belgium; ^8^ Department of Laboratory Medicine, AZ Sint-Jan Brugge-Oostende, Bruges, Belgium

**Keywords:** SARS-CoV-2, fusion peptide, spike, S2 subunit, broadly neutralizing antibodies, coronaviruses, pepscan

## Abstract

To face the continuous emergence of SARS-CoV-2 variants, broadly protective therapeutic antibodies are highly needed. We here focused on the fusion peptide (FP) region of the viral spike antigen since it is highly conserved among alpha- and betacoronaviruses. First, we found that coronavirus cross-reactive antibodies are commonly formed during infection, being omnipresent in sera from COVID-19 patients, in ~50% of pre-pandemic human sera (rich in antibodies against endemic human coronaviruses), and even in feline coronavirus-infected cats. Pepscan analyses demonstrated that a confined N-terminal region of the FP is strongly immunogenic across diverse coronaviruses. Peptide-purified human antibodies targeting this conserved FP epitope exhibited broad binding of alpha- and betacoronaviruses, besides weak and transient SARS-CoV-2 neutralizing activity. Being frequently elicited by coronavirus infection, these FP-binding antibodies might potentially exhibit Fc-mediated effector functions and influence the kinetics or severity of coronavirus infection and disease.

## Introduction

The outcome of infection with SARS-CoV-2, the cause of coronavirus disease 2019 (COVID-19), varies widely from asymptomatic to acute respiratory distress syndrome leading to death. Disease severity is correlated with advanced age, sex, genetic background and comorbidities such as diabetes, cardiovascular disease, chronic lung disease, obesity and reduced immune function (1). In young individuals, SARS-CoV-2 infection is mostly asymptomatic (2). Since adolescents and children under 5 years of age exhibit the highest infection frequency by endemic human coronaviruses (eHCoVs) (3, 4), the hypothesis has been raised that the immune response induced by these viruses might offer some levels of cross-protection against SARS-CoV-2 (4–6).

SARS-CoV-2 is the seventh coronavirus known to infect humans, besides HCoV-OC43, -229E, -NL63, -HKU1, severe acute respiratory syndrome-related coronavirus (SARS-CoV) and Middle East respiratory syndrome-related coronavirus (MERS-CoV). HCoV-229E and -NL63 belong to the *Alphacoronavirus* genus, while the *Betacoronavirus* genus encompasses HCoV-OC43 and -HKU1 (lineage A) together with the more pathogenic SARS-CoV and SARS-CoV-2 (lineage B) and MERS-CoV (lineage C). Whereas the SARS-CoV outbreak of 2003 was fully contained (7) and MERS-CoV has been limited to sporadic cases after its emergence in 2012 (8), the human alphacoronaviruses and lineage A betacoronaviruses are endemic and circulating worldwide. HCoV-OC43 is the most prevalent eHCoV and accounts, together with HCoV-229E, for about 30% of all common colds. This explains the high seroprevalence for these two viruses in the adult population (3, 4, 9–12).

Antigenic cross-reactivity against the two most immunogenic coronavirus proteins, the spike (S) and nucleocapsid (N) proteins, has been frequently reported (13–16). For SARS-CoV-2, the S and N proteins share only 38% and 32% amino acid identity with their HCoV-OC43 homologs and 33% (S) and 24% (N) with their HCoV-229E homologs (17, 18). Nevertheless, within these two proteins, strong conservation is found in short regions which were recently identified as cross-reactive epitopes (19). This conservation is plausibly related to the functions of S and N in virus replication. To be fully functional as a mediator of virus entry into host cells, the spike protein of SARS-CoV-2 requires priming: cleavage at the S1/S2 site, by host cell proteases, separates the angiotensin-converting enzyme 2 (ACE2) receptor binding S1 subunit from the membrane fusion mediating S2 subunit (20, 21). During virus entry, S2 needs additional cleavage at its S2′ site (= activation step) to liberate the fusion peptide (FP), induce membrane fusion and initiate virus replication (22–25).

Recent work with pre-pandemic sera identified the S2 subunit as a main target for coronavirus cross-reacting antibodies (14–17, 26), however this antibody response seems associated with, at best, modest virus-neutralizing activity. Ng et al. (17) did observe SARS-CoV-2 neutralization with S2 cross-reactive sera from SARS-CoV-2 uninfected individuals. In contrast, Anderson et al. (27) found barely detectable levels of SARS-CoV-2 neutralizing antibodies in pre-pandemic sera, regardless of whether the sera did or did not contain cross-reactive antibodies recognizing SARS-CoV-2 S. This lack of SARS-CoV-2 neutralizing activity was also reported for pre-pandemic sera from patients with seasonal coronavirus infection (28). Regarding the specific S2 regions targeted by cross-reactive antibodies, one recently identified region is conserved among betacoronaviruses and located in a stem helix preceding the heptad repeat 2 region (19, 29–32). A monoclonal antibody targeting this stem helix and isolated from a COVID-19 convalescent donor, was proven to inhibit S-mediated membrane fusion and possess broadly neutralizing activity against betacoronaviruses like SARS-CoV-2 (30). Even broader anti-coronavirus activity is conceivable for another S2 region that is highly conserved among alpha- and betacoronaviruses and is centered around the FP and preceding the S2′ cleavage site (19, 32, 33). An 18-mer peptide spanning this region was able to slightly reduce, by affinity depletion, the SARS-CoV-2 neutralizing activity of human sera (33), which is consistent with the presence of one or more linear neutralizing epitopes at this site. On the other hand, no SARS-CoV-2 S-pseudovirus neutralization was observed with an anti-FP antibody preparation obtained by peptide affinity purification from five convalescent sera (32). Hence, the neutralizing potential of antibodies targeting this S2 region is, thus far, unresolved.

The first aim of this study was to determine the frequency by which coronavirus cross-reactive antibodies are formed. We therefore evaluated a panel of pre-pandemic human plasma samples for cross-reactivity and cross-neutralization towards SARS-CoV-2 and, *vice versa*, a panel of COVID-19 patient sera for reactivity towards eHCoVs. Our second aim was to profile both serum panels in terms of antibody response against the most conserved part of S2. Hence, pepscan analysis was performed with a series of overlapping peptides covering residues 806-1090 of the SARS-CoV-2 spike, which encompasses the S2′ cleavage site, FP and heptad repeat 1. One specific 12-mer peptide containing the SARS-CoV-2 S2′ site and N-terminal part of the FP showed high reactivity, even with cat antibodies directed against feline coronavirus. Human antibodies specific for this spike region were isolated from a COVID-19 convalescent serum by peptide affinity chromatography, and evaluated for their ability to bind to diverse coronaviruses [i.e. SARS-CoV-2, HCoV-OC43, HCoV-229E and a non-human (i.e. feline) *Alphacoronavirus*] and neutralize their infectivity. The anti-FP antibodies proved able to efficiently bind these four distinct coronaviruses. This was paralleled with weak SARS-CoV-2 neutralizing activity. Consistent with earlier studies (14–16, 19, 33–35), our findings contribute to establish this pan-coronavirus FP region as highly immunogenic across human and animal coronaviruses. Such conservation may imply that a common immunological mechanism, that could be Fc-mediated, may be at play during coronavirus infection. Our anti-FP purified antibodies represent a relevant starting point to investigate this aspect.

## Materials And Methods

### Pre-Pandemic and Pandemic Sera, Pleural Fluid and Ascites Samples

Human plasma samples from 496 pre-pandemic blood donors collected between 2016 and 2017 were acquired through Red Cross Flanders (order numbers: CG2016 0404A, CM2016 0627B and CG2016 1219F) (36). Human serum samples were obtained from twenty hospitalized patients admitted to the Ghent University Hospital in March 2020 with RT-qPCR confirmed COVID-19 diagnosis (UZ-Ghent ethics committee approval BC-07829) (37). A written informed consent for participation to this study was obtained for the COVID-19 convalescent human serum 20Hu384.

Cat sera were submitted during the first wave of the Belgian SARS-CoV-2 pandemic (2020) for diagnosis of FeCV (cats #164, #243, #245) or SARS-CoV-2 (cat #40); pleural fluids (FIP cats #4 and #14) and ascites (FIP cats #3, #7 and #22) samples were submitted between 2004 and 2007.

All samples were incubated at 56°C for 30 min to inactivate complement.

### Cells and Viruses

PK-15 (porcine kidney) cells (ATCC CCL-33) were grown in MEM supplemented with 10% heat-inactivated (56°C, 30 min) fetal bovine serum (FBS); Huh7 cells (CLS 300156) and VeroE6 cells (ATCC CRL-1586) were grown in DMEM with 10% FBS; and HRT-18 cells (ATCC CLL-244) were grown in RPMI with 5% FBS. The feline enterocyte cell line established in our laboratory (38) was grown in collagen I-coated flasks, in a 1:1 mixture of DMEM and F12 culture media supplemented with 5% FBS and 0.1 mM non-essential amino acids (NEAA). The culture medium of these cell lines was supplemented with 100 Units/ml penicillin, 100 µg/ml streptomycin and 50 µg/ml gentamycin (only penicillin/streptomycin for Vero cells). Calu-3 cells (ATCC HTB-55) were grown in MEM with 10% FBS, 0.1 mM NEAA, 2 mM L-glutamine and 10 mM HEPES. All cells were maintained in a humidified 5% CO_2_ atmosphere at 37°C.

SARS-CoV-2 (third passage of isolate S1871-C3f) used for the neutralization test in VeroE6 cells was isolated from a nasopharyngeal swab from a COVID-19 patient (Sciensano). It was passaged three times in VeroE6 cells before nanopore sequencing (PathoSense BV, Belgium). The sequence was deposited in the GISAID database (EPI_ISL_1718321). For the neutralization assay in Calu-3 cells, a Wuhan-like SARS-CoV-2 isolate (SARS-CoV-2/Belgium/GHB-03021/2020; GISAID accession number EPI_ISL_407976; kindly donated by P. Maes, KU Leuven) was used. It was recovered from a nasopharyngeal swab of an RT-qPCR-confirmed asymptomatic case and passaged twice in Huh7 cells. The other viruses were: HCoV-OC43 (ATCC VR-759), HCoV-229E (39) and FeCV type I UCD-UU2 strain (40).

### Purification of Peptide-Specific Human Antibodies

Purification of peptide-specific human antibodies (pAbs) was performed by peptide affinity chromatography as described before (41). One mg of a 12-mer peptide (>80% purity based on HPLC-MS, JPT Peptide Technologies) was covalently coupled to a HiTrap N-hydroxysuccinimide-activated sepharose high performance column (GE Healthcare), following the manufacturer’s instructions. A blank purification procedure to wash off loosely bound peptides was performed prior use. The serum pH was adjusted to pH 7.0 with 200 mM Na_2_HPO_4_ (pH 7.0). After clarification by centrifugation, the serum was filtered (0.45 µm) and applied to the column, pre-equilibrated in 20 mM Na_2_HPO_4_ (pH 7.0). The column was subsequently washed with 20 mM Na_2_HPO_4_ buffer until the wash fractions were free of protein, based on spectrophotometry at 280 nm. Peptide-specific antibodies were rapidly eluted from the column with 0.1 M glycine (pH 2.7) and immediately neutralized in 1 M Tris (pH 8.0). Elution fractions with the highest protein concentration, as measured by spectrophotometry at 280 nm, were pooled, extensively dialyzed against PBS and stored at −70°C. The final concentrations were 163 µg/ml for pAb-PEP3, 102 µg/ml for pAb-PEP71, 274 µg/ml for pAb-PEP72 and 327 µg/ml (batch 1) or 132 µg/ml (batch 2) for pAb-PEP79. As buffer control, elution fractions devoid of proteins were collected in parallel and submitted to the same treatment.

### Expression of SARS-CoV-2 Nucleocapsid and Spike Proteins in PK-15 Cells

To create the SARS-CoV-2 N expression plasmid, we amplified the SARS-CoV-2 nucleocapsid coding sequence by RT-PCR, starting from total RNA obtained from a swab sample of a COVID-19 patient (UZGent). The spike coding sequence was amplified from a plasmid containing a synthetic codon-optimized (for porcine cells) SARS-CoV-2 S sequence (Genscript), based on the sequence obtained from the swab sample. Both cDNA sequences were cloned in a pcDNA3.1D/V5-His-TOPO vector (Invitrogen) *via* KpnI and NotI restriction sites, in frame with the V5-tag. The resulting expression plasmids were verified by nanopore sequencing (PathoSense BV).

PK-15 cells were transfected with these plasmids using Lipofectamine 3000 (Thermo Fisher Scientific) following the manufacturer’s instructions. Forty-eight hours post-transfection, cells were fixed with 4% paraformaldehyde, permeabilized with 0.01% Triton X-100 and incubated with primary antibody: anti-SARS-CoV-2 N (MyBioSource MBS569903, IgG2b); anti-S1 (Sino Biological 40592-MM57, IgG2b); anti-S2 (GeneTex 1A9, IgG1); or anti-V5-FITC antibody (IgG2a, Invitrogen). Alexa Fluor 594-conjugated goat anti-mouse IgG2b (Invitrogen, for N and S1) and Alexa Fluor 647-conjugated goat anti-mouse IgG1 (Invitrogen, for S2) were used as secondary antibodies. Nuclear staining was performed with Hoechst (10 μg/mL, Invitrogen). IgG1, IgG2a and IgG2b isotype-matched antibody controls were used in parallel. Results were analyzed by confocal fluorescence microscopy (Leica Microsystems).

For western blot analysis, lysates were prepared from approximately 1.5x10^6^ cells at 48 h post-transfection, using 200 µl of cell lysis buffer [50 mM Tris pH 7.4, 150 mM NaCl, 1% NP-40, 0.1% SDS, 5 mM EDTA and 1% protease inhibitor cocktail (Roche)]. When indicated, treatment of the lysates with PNGase F (New England Biolabs) was performed as recommended by the manufacturer. The lysates were mixed with reducing Laemmli buffer (4x), boiled for 5 min and subjected to SDS-PAGE and western blot. The bands were detected with mouse anti-V5 (GenScript) and peroxidase-labeled goat anti-mouse IgG antibodies (Dako). Alternatively, pAb-PEP3 (diluted to 0.8 µg/ml) was used as a primary antibody (for analysis of lysates of PK-15 cells expressing the SARS-CoV-2 spike), in parallel with the same dilution of elution buffer control. Here, peroxidase-conjugated goat anti-human IgG (Jackson Immunoresearch, 109-035-088; 1:10,000) was used as secondary antibody. The bands were visualized with the ECL Prime detection kit (Amersham) and pictures were taken with the ChemiDocMP Imaging System (Bio-Rad).

### Immunoperoxidase Monolayer Assays (IPMA)

#### SARS-CoV-2 N- and S-IPMA

PK-15 cells were seeded in 96-well plates at a density of 25,000 cells/well. Twenty-four hours later, they were transfected with S- or N-encoding plasmid using Lipofectamine 3000. Forty-eight hours later, the cells were washed, air-dried and stored at -20°C. Four-fold sample (i.e. pAbs or inactivated sera, plasma, pleural fluids or ascites) dilutions starting from 1:40 were prepared. IPMA was performed as previously described (42), except that 0.05% Tween-80 and a 1:200 dilution of goat peroxidase-labeled antibody to human IgG [F(ab’)_2_; Seracare, 5220-0390] were used. The IPMA titer was defined as the highest serum dilution that showed staining of cells expressing SARS-CoV-2 N or S.

Validation of the IPMAs involved 23 human sera tested in parallel in the S- and N-IPMA and in commercial serological assays for SARS-CoV-2. These assays were from EUROIMMUN (anti-SARS-CoV-2 ELISA IgG; batch E200323CP); Epitope Diagnostics (EDI, Novel Coronavirus COVID-19 IgG ELISA Kit; batch P590); Sol Scientifics (Coronavirus Disease Combined IgM/IgG Rapid Test; batch 20200301) and Dynamiker Biotechnology (Tianjin, 2019 nCOV IgG/IgM Rapid Test; batch 200301).

#### HCoV-OC43 and -229E IPMA

HRT-18 and Huh7 cells were seeded in 96-well plates at 20,000 cells/well. Two or three days post-seeding, cells were infected with HCoV-OC43 (MOI 0.05) or HCoV-229E (MOI 0.001) respectively. After 4 days for HRT-18 cells at 33°C or 2 days for Huh7 cells at 37°C, the cells were fixed and stored at -20°C. IPMA was performed as described above.

#### FeCV IPMA

Feline enterocytes were cultivated for 2 days before being washed with Ca^2+^- and Mg^2+^-enriched PBS (rPBS) containing 100 Units/ml penicillin and 100 µg/ml streptomycin. They were subsequently treated with 2 mU/ml neuraminidase (Roche, 11080725001) in rPBS for 1 h at 37°C, before being washed again and inoculated with FeCV. After 1 h adsorption, complete growth medium was added and cells were incubated for 24 h before being washed with PBS, air-dried (1 h at 37°C) and frozen at -20°C. IPMA staining was performed with serial dilutions of samples as above. Peroxidase-labelled rabbit anti-cat IgG (Nordic-MUbio; 1:100) or goat anti-human IgG antibody [F(ab’)_2_; SeraCare, 1:200) were used as secondary antibodies.

### Pepscan Analysis

Epitope mapping was performed by pepscan analysis, using sets of overlapping peptides in a peptide ELISA, as previously described (41) but with a modified blocking protocol to reduce unspecific binding. The peptide sequences (**Figure 5** and **Supplemental Figures S3, S5**) were based on the S amino acid sequences of SARS-CoV-2 (accession number YP_009724390), HCoV-OC43 (YP_009555241), HCoV-229E (NP_073551), MERS-CoV (YP_009047204), SARS-CoV (YP_009825051), FIPV type II isolate 79-1146 (YP_004070194), FeCV type II strain WSU 79-1683 (AFH58021), FeCV type I strain UU2 (ACT10948), FIPV type I strain UU3 (ACT10959) and the N sequence of SARS-CoV-2 [YP_009724397; peptide N4P5 (43)]. PEP79 corresponds to SARS-CoV-2 peptide S21P2 (33). Scrambled PEP74 corresponds to SARS-CoV-2 PEP18 with interchanged residues to maintain its amphipathicity as determined by helical wheel diagram (pepwheel, EMBOSS explorer). All peptides were provided by JPT Peptide Technologies (Berlin, Germany), as a set of BioTides that contained a hydrophilic spacer with a biotin molecule at the N-terminus and a glycine-amide at the C-terminus. To prevent interference of the blocking agent with the human sera, SuperBlock T20 Blocking Buffer (Thermo Scientific) instead of BSA was used for blocking, as recommended by JPT Peptide Technologies. All peptides were assayed without primary antibody to verify absence of non-specific binding of the secondary antibody to the immobilized peptide.

Peptides were dissolved in 100% DMSO and used to coat 96-well plates according to the instructions of the manufacturer. Briefly, streptavidin-coated plates (Thermo Fisher) were washed three times with PBS-T (PBS with 0.05% Tween-20) and coated for 1 h at room temperature (RT) with 0.1 µg biotinylated peptide diluted in peptide coating buffer (PBS-T with 40% DMSO). After four washing steps with PBS-T, plates were blocked for 30 min at RT with blocking buffer (400 µM biotin, AppliChem; 20% sucrose in PBS), air-dried and kept dry at 4°C until use. All buffers were filtered using a 0.2 µm filter.

For the pepscan, peptide-coated plates were blocked with SuperBlock T20 for 1 h at 30°C on an orbital shaker (Ohaus) at 200 rpm. Human sera as well as serum, ascites or pleural fluid from cats, were centrifuged for 10 min at 16,000 g and diluted 1:1,000 in SuperBlock T20. pAbs were diluted to 30 ng/well in SuperBlock T20. The samples were added to the peptide-coated plates for 1 h at 30°C with shaking, followed by washing with TBS-Tween 0.05% (TBS-T). Next, a 1:5,000 dilution in SuperBlock T20 of peroxidase-labeled goat anti-human IgG (Jackson ImmunoResearch) or a 1:2,000 dilution of peroxidase-labeled rabbit anti-cat IgG (Nordic-MUbio) was added. After 1 h incubation at 30°C with shaking, the plates were washed with TBS-T and TMB substrate (3,3’,5,5’-Tetramethylbenzidine Substrate Kit, Thermo Scientific) was added. The reaction was stopped after 7 min at 30°C, by adding 100 µl of a 1:4 dilution of sulfuric acid, followed by optical density (OD) measurement at 450 nm (ThermoLabsystems Multiskan RC).

### Analysis of pAb Binding by Surface Plasmon Resonance

To evaluate the interaction between the pAbs and the peptides or the spike protein of SARS-CoV-2, surface plasmon resonance (SPR) analysis was performed on a Biacore T200 instrument (GE Healthcare). Anti-Human IgG (Human Antibody Capture Kit, Cytiva) was immobilized on a CM5 chip and used to capture the pAbs or control antibodies. As analytes, we used 1:100 diluted peptides or recombinant SARS-CoV-2 spike protein, i.e. full-length trimeric S protein from NativeAntigen (REC31871) or its separate domains from Sino Biological (S2, 40590-V08H1; S1, 40591-V08H; or RBD, 40592-VNAH). Analytes were injected in HBS-EP+ buffer (10 mM HEPES, 150 mM NaCl, 3 mM EDTA and 0.05% v/v Surfactant P20) for 2 min at a flow rate of 30 μl/min, and the dissociation was monitored for 2 min. The chip was finally regenerated with 3M MgCl_2_. Commercial antibodies anti-S2 (Sino Biological, 40590-D001) and REGN10987 (kindly donated by PharmAbs, KU Leuven) were included as controls.

### Neutralization Assays With Different Coronaviruses

#### Neutralization Test With SARS-CoV-2 (S1871-C3f) in Vero E6 Cells

Serial two-fold sample dilutions (1:40 - 1:5120) in DMEM with 2% FBS, 100 units/ml penicillin and 100 µg/ml streptomycin were incubated for 1 h at 37°C with SARS-CoV-2 (isolate S1871-C3f) at 100 TCID_50_/well. These mixtures were inoculated on confluent monolayers of VeroE6 cells in 96-well plates, and incubated for 48 h at 37°C. Next, cells were fixed by 30 min incubation at 4°C with cold 4% paraformaldehyde in PBS and washed twice with PBS. The cells were permeabilized with 0.2% Triton X100 in PBS for 30 min at RT. After two washes with PBS, cells were incubated for 1 h at RT in a 1:2,000 dilution in ELISA buffer (0.1% Tween 80, 10% horse serum in PBS) of anti-SARS-CoV-2 N antibody (40143-MM08, SinoBiological). After three washes with washing buffer (0.05% Tween-80 in PBS), cells were incubated for 1 h at RT with a 1:1,000 dilution of peroxidase-labeled goat anti-mouse immunoglobulin (Bio-Rad 172-1011) in ELISA buffer. After three washes with washing buffer, KPL True Blue peroxidase substrate (VWR) supplemented with 0.03% H_2_O_2_ was added and incubated for 30 min at RT, before two washes with water to remove the substrate. The Reed-Muench method was used to calculate the neutralizing antibody titer that reduced the number of infected wells by 50%. A positive, negative and no serum control was included in each experiment.

#### SARS-CoV-2 Neutralization Test in Calu-3 Cells

Serial 2.5-fold dilutions of peptide-purified antibodies (starting from 1:2), control serum (starting from 1:50) or control antibody (starting from 10 µg/ml) were incubated for 1 h at 37°C with SARS-CoV-2 (SARS-CoV-2/Belgium/GHB-03021/2020). The mixtures were then added to Calu-3 cells at a final MOI of 100 TCID_50_/well and incubated at 37°C. The infection medium consisted of MEM supplemented with 0.1 mM NEAA, 2 mM L-glutamine, 10 mM HEPES, 100 U/mL of penicillin, 100 µg/ml of streptomycin, 0.2% FCS and 0.3% BSA. At 2 h p.i., the cells were washed with infection medium and further incubated at 37°C. At 24 h p.i., the number of viral genome copies in the supernatant was determined by qRT-PCR using the CellsDirect One-Step RT-qPCR kit (Invitrogen) as described before (44), with primers and probe for the SARS-CoV-2 N gene (US CDC 2019-nCoV_N1; IDT) and N plasmid standard (2019-nCoV_N positive control plasmid; IDT). At 72 h p.i., virus replication was quantified by immunofluorescence staining for dsRNA, using the J2 dsRNA antibody (SCICONS) combined with Hoechst staining as previously described (45). A monoclonal SARS-CoV-2 neutralizing anti-S1 antibody (SinoBiological 40592-R001) was included as reference.

#### HCoV-OC43

serial two-fold sample dilutions (1:8 up to 1:16,384) in DMEM supplemented with 5% FBS, 100 Units/ml penicillin and 100 µg/ml streptomycin were incubated for 1 h at 37°C with 500 TCID_50_/well of HCoV-OC43 in 96-well plates. HRT-18 cells were added at 20,000 cells/well and incubated for 4 days at 33°C. Plates were washed with PBS, fixed, and IPMA staining was performed as described above, using an antibody directed against the HCoV-OC43 nucleocapsid (mAb9013, Sigma-Aldrich; 1:1,000) and peroxidase-labeled goat anti-mouse IgG (Dako, P0447; 1:1,000).

#### FeCV

Seventy TCID_50_ of FeCV were mixed with serial two-fold sample dilutions in culture medium without FBS. Feline enterocytes were cultivated for 2 days at 37°C before being washed with PBS supplemented with MgCl_2_, CaCl_2_ and penicillin/streptomycin. After trypsinization, 40,000 cells per well were added to the mixtures of virus and sample in collagen I-coated 96-well plates. After 1 h adsorption, complete medium was added and cells were incubated for 3 days at 37°C before being washed and fixed for IPMA staining. Anti-FeCV nucleocapsid antibody 10A12 (46) and peroxidase-labeled goat anti-mouse immunoglobulin (Dako P0447) were used for staining.

### Statistical Analyses

Statistical analyses were performed with GraphPad Prism 9.1.2 (GraphPad Software, San Diego, California, USA). For the longitudinal study on sera from hospitalized COVID-19 patients, IPMA titers [from the 13 patients (A, B, D, H, I, J, K, M, P, Q, R, S, and T) for which such data were available] were compared from one week to the next. An ordinary one-way ANOVA, followed by Tukey’s multiple comparisons test. When the adjusted *P* value was less than 0.05, the difference was considered statistically significant. A Pearson’s correlation matrix was established to analyze the correlation, for patients A-O, between SARS-CoV-2 neutralizing antibody titer and IPMA-based antibody titers against SARS-CoV-2 N and S, HCoV-OC43 and HCoV-229E. Pearson correlation coefficient (*r*) and two-tailed p-values were calculated for all pairs (n=44). For the SARS-CoV-2 neutralization test in Calu-3 cells, statistical significance of differences between the treated samples and untreated virus control was analyzed by multiple unpaired t-tests, corrected for multiple comparisons using the Holm-Šídák method.

## Results

### Detection of Anti-Coronavirus IgG in Human Sera

**Figure 1:**
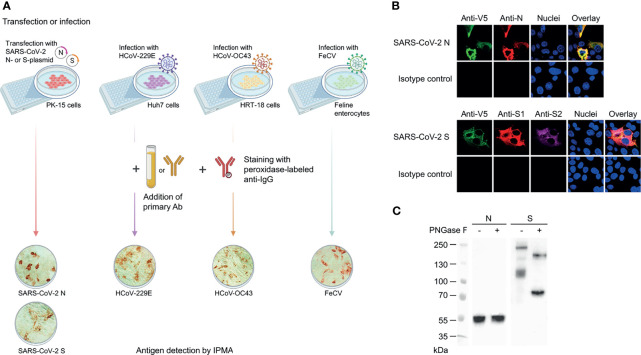
IPMA assay for determination of antibody titers against SARS-CoV-2 S or N, or HCoV-229E, HCoV-OC43 and FeCV. **(A)** Assay principle and representative images of positive IPMA staining. For SARS-CoV-2, we used PK-15 cells transfected with an expression plasmid for V5-tagged S- or N-protein. For HCoV-229E, HCoV-OC43 and FeCV, we used virus-infected Huh7 cells, HRT-18 cells and feline enterocytes, respectively. Either serum, plasma, pleural fluid, ascites or purified antibody were used as primary antibodies; peroxidase-labelled goat anti-human or rabbit anti-cat IgG were used as secondary antibodies. **(B)** Staining with commercial antibodies confirmed SARS-CoV-2 N and S expression in transfected PK-15 cells, as evident from strong colocalization of anti-V5 and anti-N staining (top panels); and anti-V5, anti-S1 and anti-S2 staining (bottom panels). **(C)** Western blot showing proper expression of N and S in the transfected PK-15 cells, with full-length S undergoing S1/S2 cleavage and *N*-glycosylation, as evident from PGNase F treatment on the cell lysates.

To quantify coronavirus cross-reactive antibodies in human sera, we developed immunoperoxidase monolayer assays (IPMA) in cells that were either infected with virus (HCoV-OC43 or HCoV-229E) or transfected with an expression plasmid for V5-tagged SARS-CoV-2 S or N protein, which avoided biosafety level-3 manipulation of live SARS-CoV-2 (**Figure 1A**). We first verified proper expression of these two proteins in porcine PK-15 cells. This was evident from immunofluorescence with commercial antibodies, showing colocalization of staining for V5 and N and for V5, S1 and S2 (**Figure 1B**). Also, western blot (**Figure 1C**) confirmed formation of full-length S protein (MW ~216 kDa) in combination with V5-tagged S2 subunit (~110 kDa) resulting from S1/S2 cleavage. After *N-*glycan removal by PNGase F, these bands shifted to the expected MW of 180 kDa (uncleaved S) and 80 kDa (S2). For the non-glycosylated N protein (~52 kDa), PNGase F treatment had no effect, as expected. Hence, the transfected PK-15 cells proved suitable for convenient IPMA detection of anti-S and anti-N IgG in human sera. To validate this, we analyzed a series of 23 serum samples from seven COVID-19 patients, hospitalized during the first pandemic wave in Belgium in 2020 (SARS-CoV-2 RT-qPCR positive patients I-O; sera taken between day 2 and 18 after onset of symptoms). The IPMA titer was defined as the highest serum dilution yielding red cell staining (illustrated in **Figure 1A**). Parallel testing with four commercial SARS-CoV-2 IgG detection assays (**Supplemental Figure S1**) showed that samples with an N- or S-IPMA titer ≥640 were all positive in the commercial tests (except for patient O at day 11) and, conversely, samples with IPMA titers <40 were negative or borderline in the commercial tests. For the sera having an N- or S-IPMA titer between 40 and 160, the commercial tests were less consistent, with some yielding a negative and others yielding a borderline result.

### Antibodies Against HCoV-OC43 and HCoV-229E Are Ubiquitous in Pre-Pandemic Samples and a Fraction of These Show Spike Binding Yet Non-Neutralizing Activity Towards SARS-CoV-2

**Figure 2:**
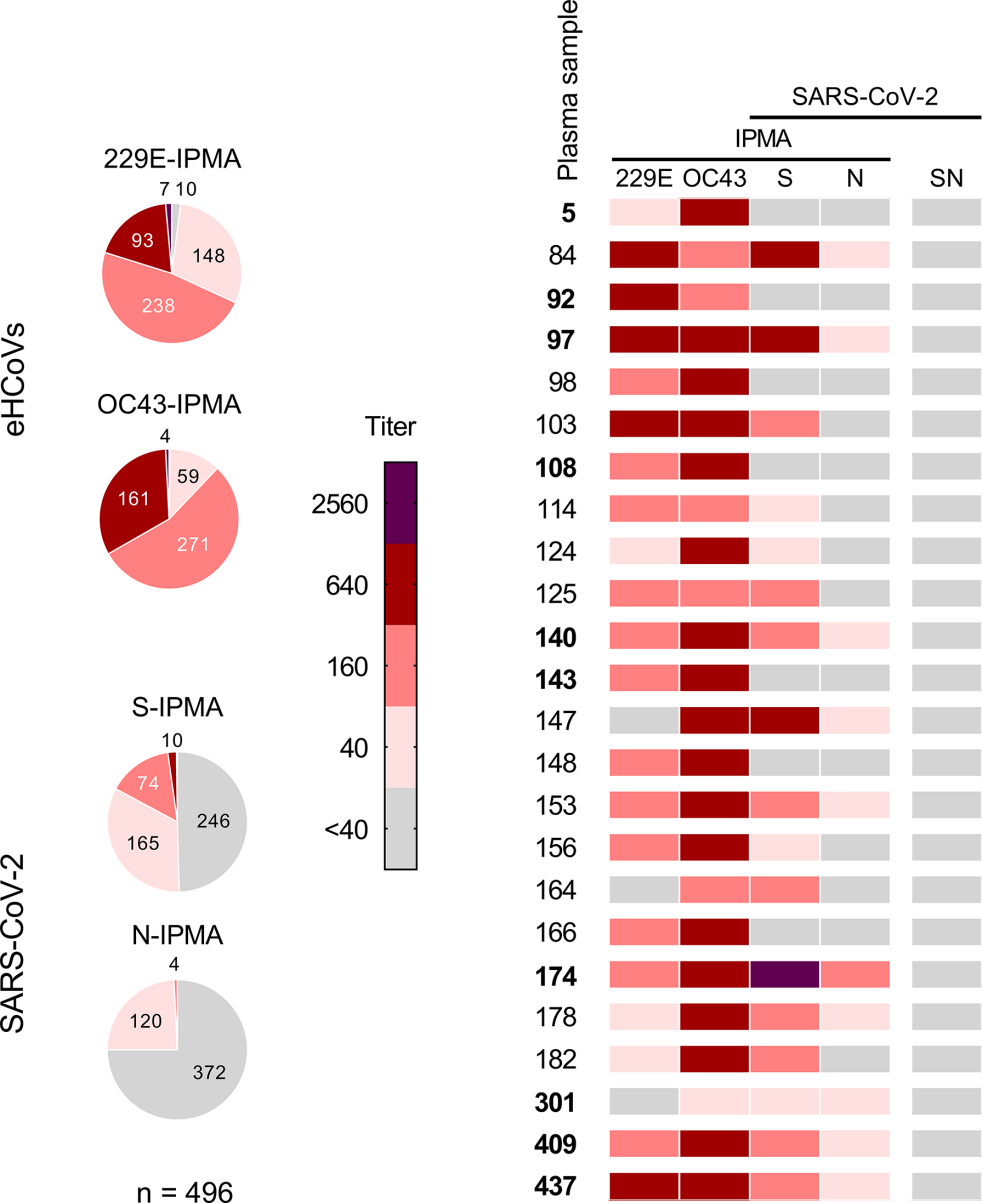
Antibody titers in pre-pandemic human plasma samples (N = 496; period 2016-2017). The IPMA titers for antibodies against HCoV-229E, HCoV-OC43, SARS-CoV-2 S and SARS-CoV-2 N of a panel of 496 pre-pandemic plasma samples are given in pie charts (left). For each slice, the absolute number of samples is added, except for two samples (having a titer <40 for OC43-IPMA or 2560 for S-IPMA). Twenty-four of these samples were selected and analysed for their SARS-CoV-2 neutralizing titer, as shown in the heatmap (right).

First, we addressed whether eHCoVs elicit cross-reacting antibodies and whether these antibodies can neutralize SARS-CoV-2, as seen in another study (17). To obtain human sera with high anti-eHCoV antibody titers, the IPMA assays were applied to a panel of 496 samples from pre-pandemic adult blood donors, collected in Belgium in 2016 and 2017. Only one (0.2%) and ten (2%) of these samples were negative (titer <40) in the HCoV-OC43- and HCoV-229E-IPMA, respectively (pie charts in **Figure 2**). Fifty-five percent and 48% of the samples had an IPMA titer of 160 for HCoV-OC43 and HCoV-229E, respectively. The fraction having a titer ≥640 was 33% for HCoV-OC43 and 20% for HCoV-229E. To detect cross-reacting antibodies, we submitted the entire sample panel to SARS-CoV-2 S- and N-IPMA. In the S-IPMA, 2.2% showed a titer ≥640 and 48.2% showed low positivity (titer 40-160). The positivity ratio was lower in the N-IPMA, as none of the samples contained an N-IPMA titer ≥640, and only 25% had a low titer of 40-160 (**Figure 2**). These low titers were found to be inconsistently positive when compared to commercial tests (**Supplemental Figure S1**).

Based on the above results, we selected 24 plasma samples for evaluation by SARS-CoV-2 neutralization assay. The selection contained 17 samples with IPMA titers ≥160 for both HCoV-229E and HCoV-OC43, and among this subset, 9 samples had a SARS-CoV-2 S-IPMA titer ≥160 (heatmap in **Figure 2**). Neither of the 24 samples was able to neutralize SARS-CoV-2 virus (seroneutralization titer <40). This was even seen for plasma #174, which had an S-IPMA titer as high as 2560 and an HCoV-OC43-IPMA titer of 640.

Hence, analysis of this pre-pandemic cohort demonstrated that many individuals carried relatively high titers of anti-eHCoV antibodies prior to the SARS-CoV-2 pandemic, with antibodies against HCoV-OC43 being particularly prevalent. About 50% of the pre-pandemic samples showed a low to intermediate level of cross-reactivity against SARS-CoV-2 S, however these spike-binding antibodies appeared devoid of virus-neutralizing activity.

### SARS-CoV-2 Infection Elicits Antibodies With Cross-Reactivity Towards HCoV-OC43

**Figure 3:**
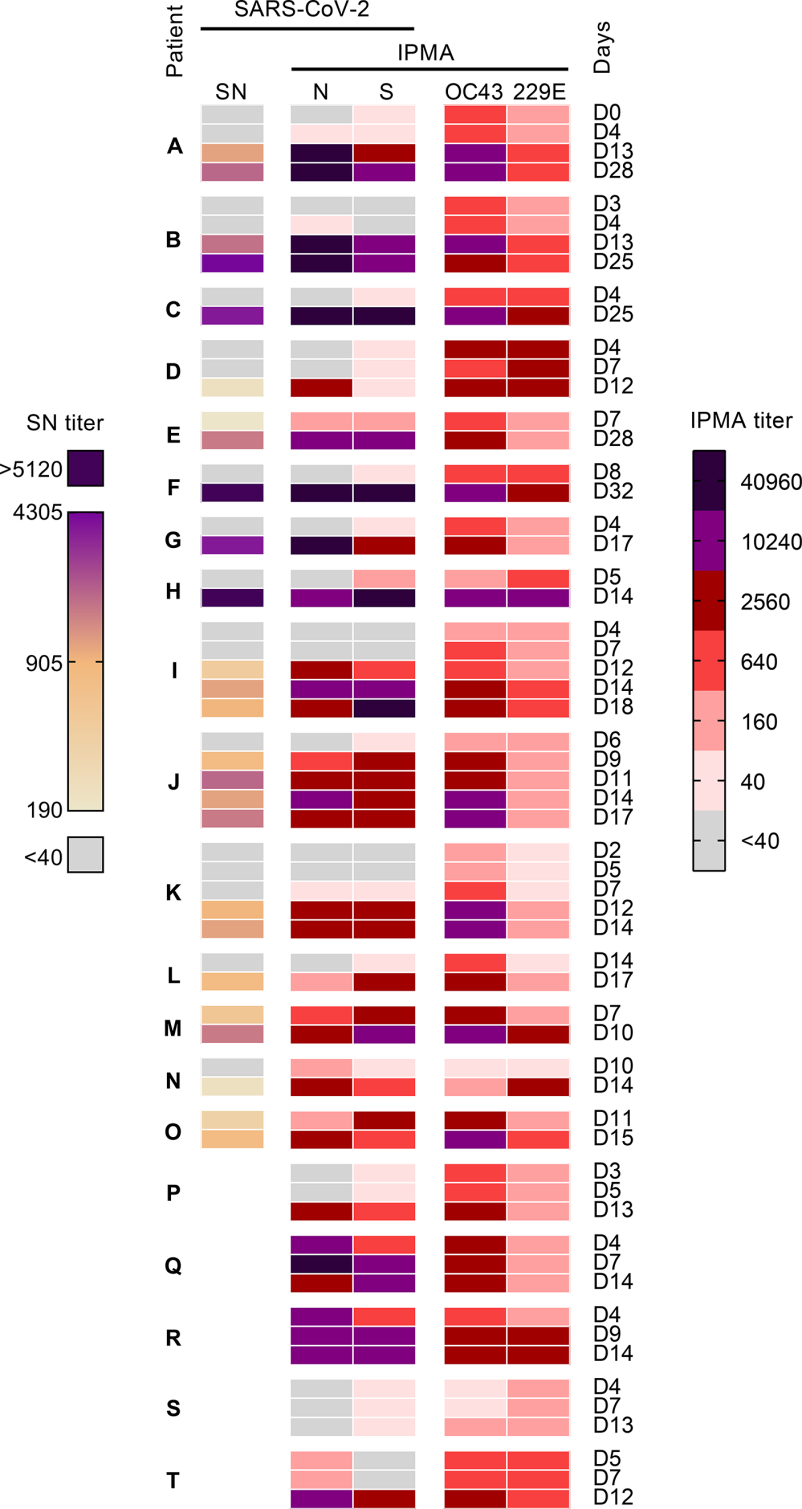
Antibody titers in sera from 20 hospitalized COVID-19 patients, from day 0 up to day 32 post-symptom onset. For 15 patients **(A–O)**, SARS-CoV-2 neutralizing antibody titers (SN titer) were determined, together with IPMA-based antibody titers against SARS-CoV-2 N and S, HCoV-OC43 and HCoV-229E. Sera from patients **(P–T)** were only partially analysed (lower part of the heatmap).

As expected and in sharp contrast to the pre-pandemic samples, SARS-CoV-2 neutralizing antibodies proved abundant in a cohort of 15 hospitalized COVID-19 patients. Specifically, among a panel of 44 sera (patients A-O, samples taken between day 0 and day 32 after symptom onset), 26 samples were positive for SARS-CoV-2 neutralizing antibodies with titers ranging from 190 up to over 5120 (**Figure 3**). These sera were also positive by IPMA, since a titer ≥640 was measured in 23 (N-IPMA) and 24 (S-IPMA) out of 26 samples, while the remaining 3 (N-IPMA) and 2 (S-IPMA) samples had low titers (40–160). Conversely, all serum samples with S- or N-IPMA titers ≥640 were positive for SARS-CoV-2 neutralizing activity, validating further our novel IPMAs (**Figure 3**).

**Figure 4:**
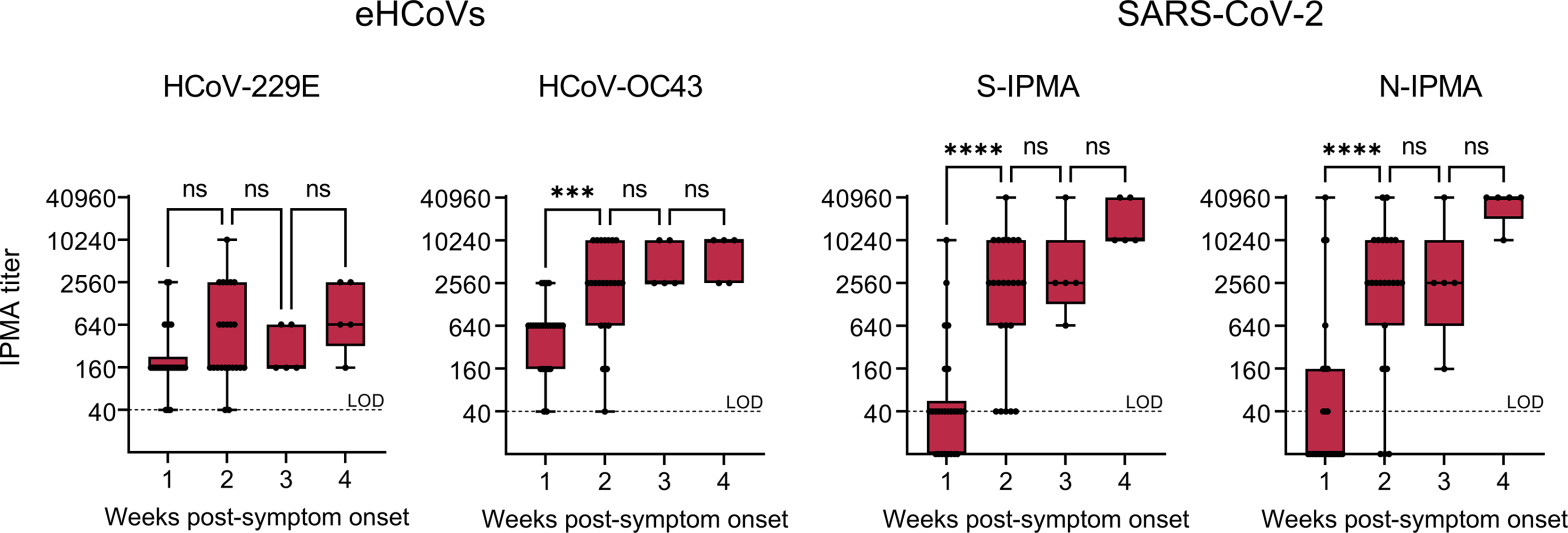
Antibody titers in sera from 20 COVID-19 patients across the first four weeks post-symptom onset. Antibody titers based on IPMA for HCoV-229E, HCoV-OC43, SARS-CoV-2 S (S-IPMA) and N (N-IPMA). The boxes extend from the 25th to 75th percentiles, while the whiskers range from the minimum to maximum values. Each individual point is plotted, the line in the middle (when applicable) is the median. Not significant (ns), P > 0.05; ***P ≤ 0.001; ****P ≤ 0.0001 (ordinary one-way ANOVA followed by Tukey’s multiple comparisons test, one week versus the next).

To investigate whether the infection elicits coronavirus cross-reactive antibodies, we analyzed a slightly expanded serum panel (twenty patients A-T; 59 serum samples taken between day 0 and day 32 after symptom onset). All COVID-19 patients had detectable antibody levels (i.e. IPMA titer >40) for HCoV-229E or HCoV-OC43 at onset of disease, before SARS-CoV-2 anti-N and -S antibodies became detectable (**Figure 4**). Between the first and second week after symptom onset, the IPMA titer significantly increased for HCoV-OC43 (P=0.0008) but not for HCoV-229E (P=0.2035). This coincided with an even more significant (P<0.0001) and expected increase in the SARS-CoV-2 N- and S-IPMA titers (**Figure 4**). Since it is highly unlikely that all these COVID-19 patients became concomitantly co-infected with HCoV-OC43, this increase in anti-HCoV-OC43 response indicates that SARS-CoV-2 elicits antibodies that are cross-reactive to conserved betacoronavirus antigens. This might involve a recall of pre-existing anti-HCoV-OC43 memory B cells (47).

This cross-reactivity was also evident from Pearson correlation analysis on the data from patients A-O. A clear correlation (**Supplemental Figure S2**) was observed not only between SARS-CoV-2 neutralizing antibody titer (SN) and S- or N-IPMA titers (*r*=0.89 and 0.93), but also between SARS-CoV-2 SN titer and HCoV-OC43 IPMA titer (*r*=0.79). For the analysis of SN titer versus IPMA titer for HCoV-229E, the correlation was much weaker (*r*=0.37).

### Antibodies Recognizing the Conserved S2′ Cleavage Site and N-Terminal Region of the Fusion Peptide Are Elicited in COVID-19 Patients

**Figure 5:**
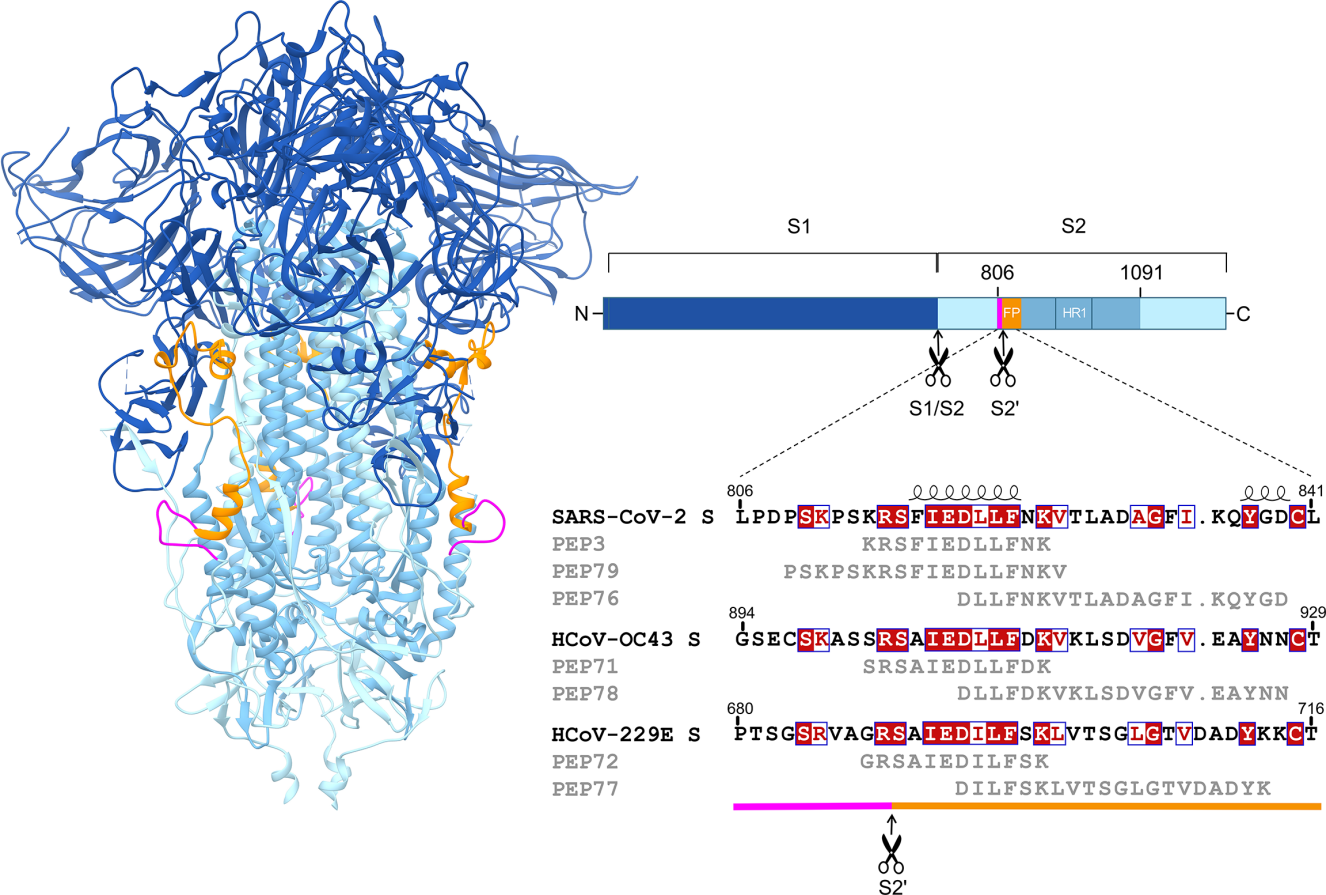
Alignment of selected PEPs with the viral spike sequences. The peptides were derived from a part of the SARS-CoV-2 spike S2 domain (residues 806-1091), encompassing the S2′ cleavage site, fusion peptide and heptad repeat 1. On the left, the structure of the SARS-CoV-2 spike in closed conformation is shown (PDB 6ZGE). The right panel shows an alignment of the relevant S2 sequences of SARS-CoV-2 (NCBI accession number YP_009724390), HCoV-OC43 (YP_009555241) and HCoV-229E (NP_073551), as well as the corresponding peptides. Sequence similarities from Clustal Omega-aligned sequences were rendered using ESPript 3.0. Shown in red shading: fully conserved residues; in red font: residues are similar according to physicochemical properties; and boxed in blue: ≥70% similarity. Secondary structure depiction shown on top was derived from PDB 6ZGE.

Our analyses of pre-pandemic samples and COVID-19 sera align with other reports (13) that cross-reactive antibodies are formed in the context of human coronavirus infections, hence identifying the epitopes for this broad recognition is of high interest. We decided to focus on a domain of the spike protein that is particularly well-conserved among SARS-CoV-2 and endemic alpha- and betacoronaviruses. This region spans residues 806-1091 in SARS-CoV-2 S, is located in the S2 subunit and encompasses the S2′ cleavage site, putative fusion peptide (FP) and heptad repeat 1 (**Figure 5**). This fusion peptide region was reported to be antigenic and elicit cross-reactivity (14–16, 19, 34, 35). To dissect the role of specific epitopes within this region, we performed pepscan analyses on our two panels of human sera. Hence, we designed a series of 70 overlapping biotinylated dodecapeptides covering this region (PEP 1-70; full sequence details are given in **Supplemental Figure S3**), with an offset of 4 and an overlap of 8 amino acids. Besides these overlapping peptides, we designed 12-mer peptides based on the spike of HCoV-OC43 (PEP71) and HCoV-229E (PEP72) (**Figure 5**). PEP71 and PEP72 are homologous to SARS-CoV-2 PEP3 and encompass the S2′ cleavage site and N-terminal part of the FP. A fragment of SARS-CoV S, containing an antigenic determinant corresponding to residues 821-846 in SARS-CoV-2 S, was found to induce neutralizing antibodies towards SARS-CoV S-pseudovirus (48), though only in a subset of the immunized mice or rabbits. We therefore also synthetized three longer (20-mer) peptides covering most of this region in SARS-CoV-2 (PEP76), HCoV-229E (PEP77) and HCoV-OC43 (PEP78) (**Figure 5**). To check the specificity of the pepscan analysis, a control peptide (PEP74, scrambled sequence of PEP18, **Supplemental Figure S3B**) was included as negative control.

**Figure 6:**
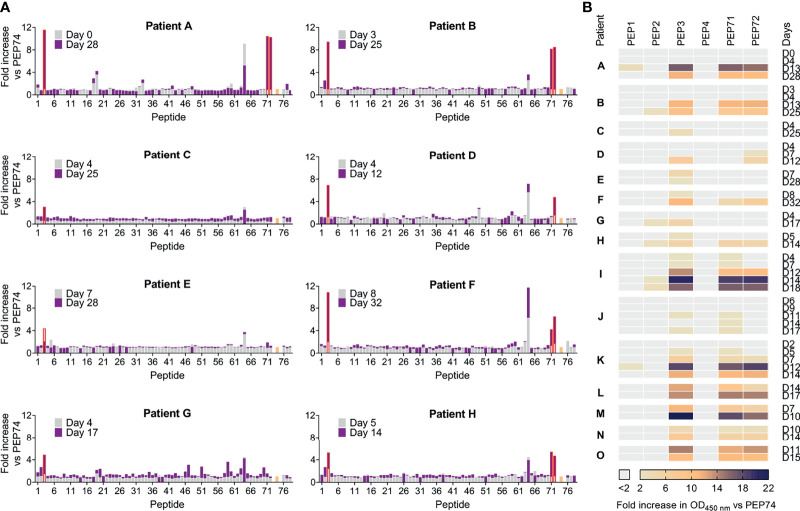
Pepscan analysis on sera from hospitalized COVID-19 patients. **(A)** Paired sera from eight hospitalized COVID-19 patients, collected at an early and late time-point post-symptom onset, were analysed by pepscan. Bar graphs show the fold increase in OD_450 nm_ value versus control peptide PEP74 (colored in salmon). The series of 76 peptides consisted of: 70 overlapping peptides derived from SARS-CoV-2 (PEP1-PEP70 and PEP76); 2 peptides derived from HCoV-OC43 (PEP71 and -78); 2 peptides derived from HCoV-229E (PEP72 and -77); and PEP74: negative control peptide (scrambled). **(B)** Serum samples from additional time points (ranging from day 0 up to day 32 post-symptom onset) and seven additional hospitalized COVID-19 patients were subjected to pepscan. The fold increase in OD_450 nm_ value for PEP1-4 (derived from SARS-CoV-2 S), PEP71 (HCoV-OC43 S) and PEP72 (HCoV-229E S) is shown versus negative control peptide PEP74.

The panel of sera from COVID-19 patients was used to monitor the levels of antibodies recognizing these peptides and their evolution during the course of COVID-19 disease. Paired sera from eight patients (A-H; **Figure 6A**), taken at two successive time points, were submitted to full pepscan analysis with 76 peptides. Four peptides, i.e. PEP3, PEP64, PEP71 and PEP72, showed markedly higher reactivity than the others (**Figure 6A**). PEP64 reactivity was present at both time points, in all sera. In a preliminary pepscan to exclude non-specific binding, PEP64 was shown to bind the secondary goat antibody in the absence of primary (human serum) antibody and thus represent an unspecific IgG binder. Overall, only PEP3 (highlighted in red in **Figure 6A**), PEP71 and PEP72 gave a substantial OD_450 nm_ increase at time point 2 (in purple), relative to time point 1 (in grey). All patients showed increased PEP3-reactivity at the second time point, except for patient E whose PEP3 reactivity was already seen at the first time point and decreased thereafter. In four patients (A, B, F, H), high PEP3 positivity at the second time point appeared to coincide with reactivity against PEP71 (HCoV-OC43) and PEP72 (HCoV-229E). For the long peptides PEP76 (SARS-CoV-2), PEP77 (HCoV-229E) and PEP78 (HCoV-OC43), which lack the first six residues of PEP3, PEP71 and PEP72, no reactivity was seen at either time point.

We then selected seven peptides (PEP1, PEP2, PEP3, PEP4, PEP71, PEP72, PEP74) to perform the pepscan on sera from seven additional COVID-19 patients, giving a total of 15 (patients A-O, **Figure 6B**). All patients showed a rise in PEP3-reactivity in the course of infection and 12 of them also reacted towards PEP71 and/or PEP72, indicating coronavirus cross-reactivity. Besides, in four patients, minor reactivity against PEP2 became apparent at the latest time points. To conclude, antibodies recognizing PEP3 are commonly elicited by SARS-CoV-2, confirming previous reports on the antigenicity of the FP region (14–16, 19, 33–35).

### PEP3-Reactive Antibodies Are Also Present in Some Pre-Pandemic Human Plasma Samples and Samples From Feline Coronavirus-Infected Cats

**Figure 7:**
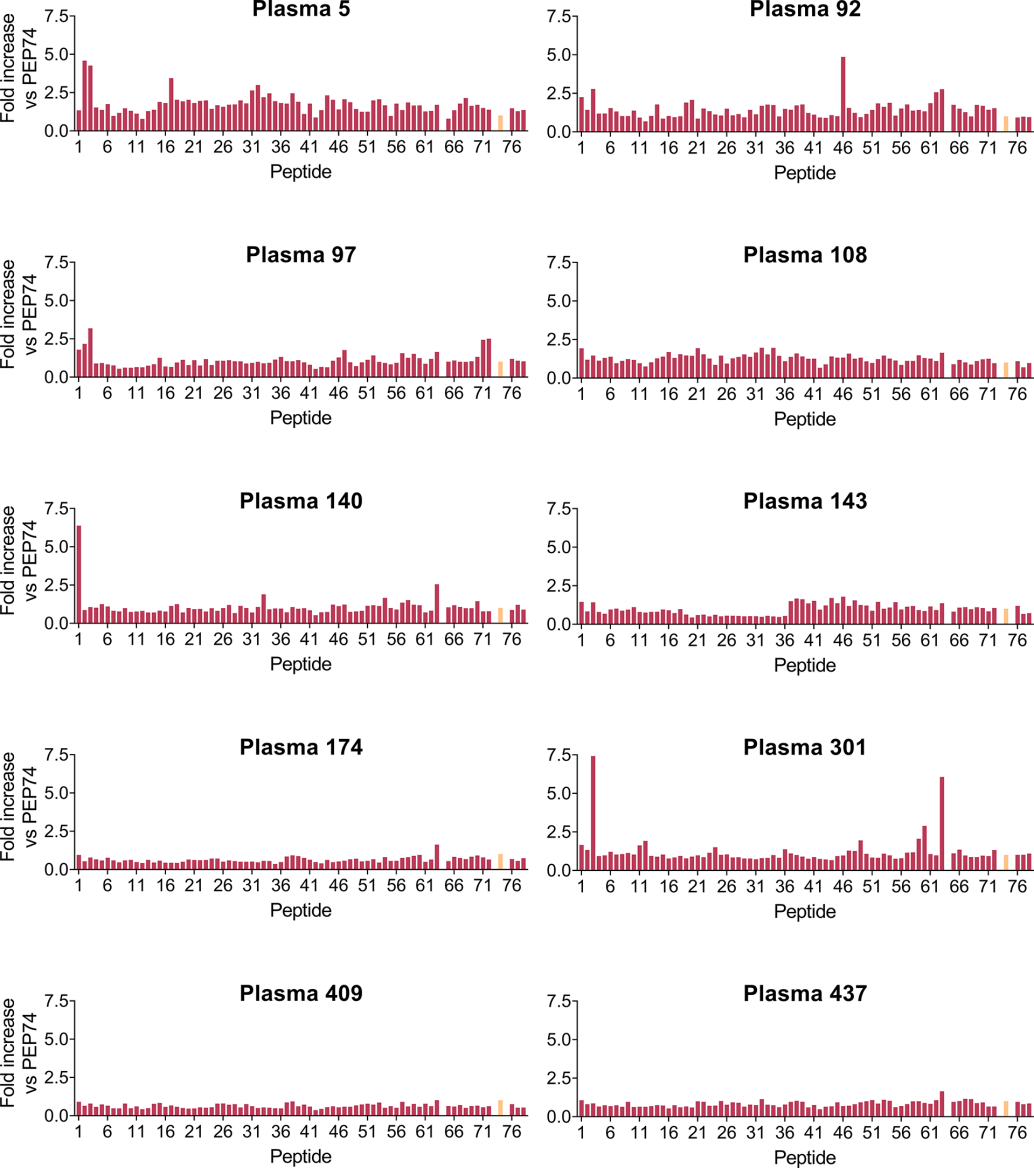
Pepscan analysis on ten pre-pandemic plasma samples. Bar graphs show the fold increase in OD_450 nm_ value versus control peptide PEP74 (colored in salmon). The series of 76 peptides consisted of: 72 overlapping peptides derived from SARS-CoV-2 (PEP1-PEP70 and PEP76); 2 peptides derived from HCoV-OC43 (PEP71 and -78); 2 peptides derived from HCoV-229E (PEP72 and -77); and PEP74: negative control peptide (scrambled). PEP64 (unspecific binder) was removed for clarity.

Next, we performed the full pepscan analysis on ten pre-pandemic human plasma samples (**Figure 7**), selected as representative for the large (n=496) panel (see **Figure 2**), based on their positive or negative result in the SARS-CoV-2 S- or N-, HCoV-OC43 and HCoV-229E IPMA analyses (see above). Interestingly, PEP3-reactivity (defined as >2-fold increase in OD_450 nm_ vs PEP74) was found in 4 out of 10 samples (**Figure 7**, #5, #92, #97 and #301), whereas other peptides were only sporadically recognized (for instance PEP1, PEP46 and PEP63). PEP3-reactivity appeared not associated with a positive result in SARS-CoV-2 S-IPMA (compare **Figure 7** with **Figure 2**; the selected 10 samples are shown in bold here). For example, PEP3-reactivity was comparable for plasma #5, #92, #97 and #301 (~3- to 7-fold increase in OD_450 nm_ vs PEP74); #5, #92 and #301 had no (<40) or low (=40) S-IPMA titer, but #97 had a titer of 640 (**Figure 2**). Also, PEP71- and PEP72-reactivity was not observed (except for #97 that recognized PEP3, PEP71 and PEP72), despite positive IPMA titers for HCoV-OC43 and -229E in almost all samples. Plasma #174 gave a positive result in the four IPMAs but showed no reactivity against PEP3 and, reciprocally, #301 was not or weakly responsive in the four IPMAs but showed the highest PEP3-reactivity (~7-fold increase in OD_450 nm_ vs PEP74) among all ten samples. Similar to what was observed in the COVID-19 patient sera, these pre-pandemic plasma samples showed no reactivity for long peptides PEP76 (SARS-CoV-2), PEP77 (HCoV-229E) and PEP78 (HCoV-OC43), which partly correspond to a SARS-CoV immunogenic region (48). Taken together, this pepscan analysis demonstrated clear reactivity of some pre-pandemic human plasma samples towards the PEP3 sequence of SARS-CoV-2 S or its homologue from HCoV-229E or -OC43. The lack of correlation to anti-coronavirus antibody titers measured by IPMA could be related to various factors, such as: recognition, in the S-IPMA assay, of betacoronavirus cross-reactive antibodies (30) targeting an S2 region outside the sequence covered by our peptide library; recognition, in the HCoV-229E and -OC43 IPMAs, of antibodies binding to other viral proteins than the spike; or recognition, in the IPMAs, of antibodies against conformational epitopes that are not detectable by pepscan analysis.

**Figure 8:**
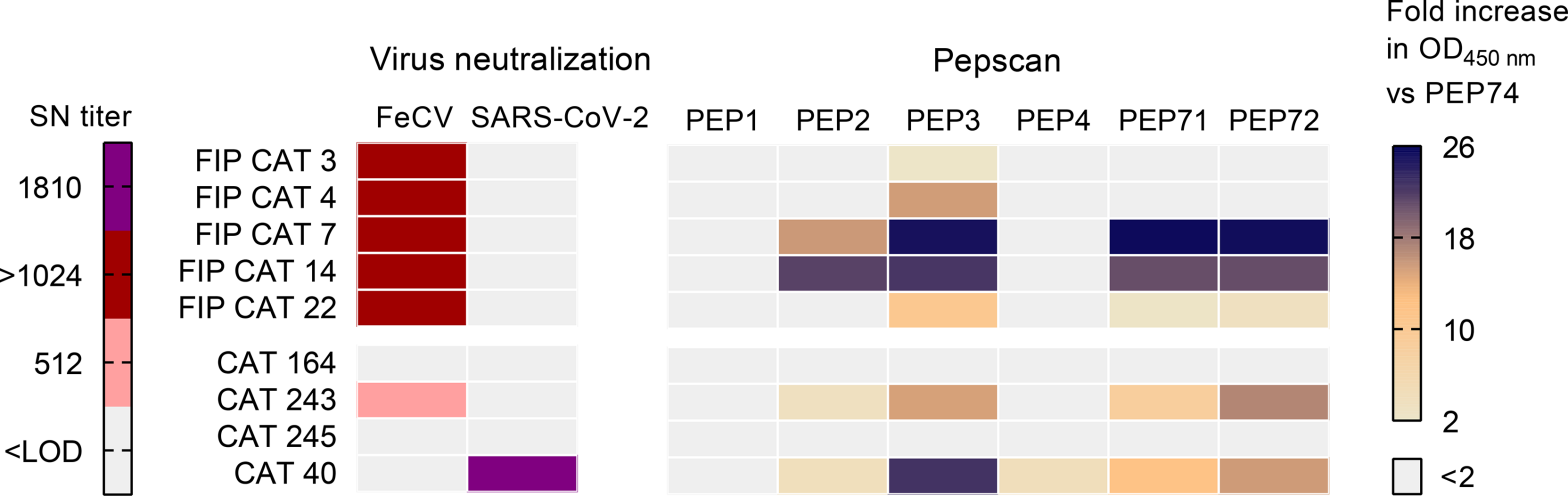
Reactivity of cat samples in seroneutralization assays and pepscan. Samples from FIP cats (cats 3, 4, 7, 19 and 22) were collected between 2004 and 2007, the other samples were collected in 2020 for diagnosis of FeCV (cats 164, 243 and 245) or SARS-CoV-2 (cat 40). The virus-neutralizing activity (left) was determined by seroneutralization (SN) assay against FeCV in feline enterocytes, and SARS-CoV-2 in VeroE6 cells. The limit of detection (LOD) was a 1:8 (FeCV) or 1:40 dilution (SARS-CoV-2). A pepscan (right) was performed using PEP1-4 (derived from SARS-CoV-2 S), PEP71 (HCoV-OC43 S) and PEP72 (HCoV-229E S). The fold increase in OD_450 nm_ value is shown versus negative control peptide PEP74.

Having observed that the well-conserved PEP3 sequence is widely immunogenic upon human coronavirus infection, we wondered whether this might also apply to a non-human coronavirus infection. To address this, we selected feline enteric coronavirus (FeCV) since it is ubiquitous in the cat population (49). This virus causes mostly mild enteritis but can persist over a year and lead to mutational variants causing a potentially chronic and fatal disease known as feline infectious peritonitis (FIP) (50, 51). Another argument to investigate cats is their known susceptibility to SARS-CoV-2 (52). Samples from nine cats were submitted to IPMA, virus-neutralization and pepscan analyses (**Figure 8**). Strong FeCV-seropositivity (IPMA titer ≥2560 and neutralization titer ≥512) was found in five pre-pandemic FIP cats, while low antibody response for this virus was detected in one out of four cats sampled during the SARS-CoV-2 pandemic. Another animal in this group, seronegative for FeCV, had been infected by SARS-CoV-2, given its high titer of SARS-CoV-2 neutralizing antibodies. In the pepscan, all FeCV-seropositive samples showed reactivity to PEP3, and most reacted also to PEP71 and PEP72. This reactivity was absent in the two cat sera devoid of anti-FeCV and -SARS-CoV-2 antibodies. Hence, not only SARS-CoV-2 but also FeCV elicits antibodies that cross-react with PEP3, PEP71 and PEP72.

### Affinity Purification With PEP3 and Homologous Peptides Yields Antibodies With Pan-Coronavirus Binding Activity

**Figure 9:**
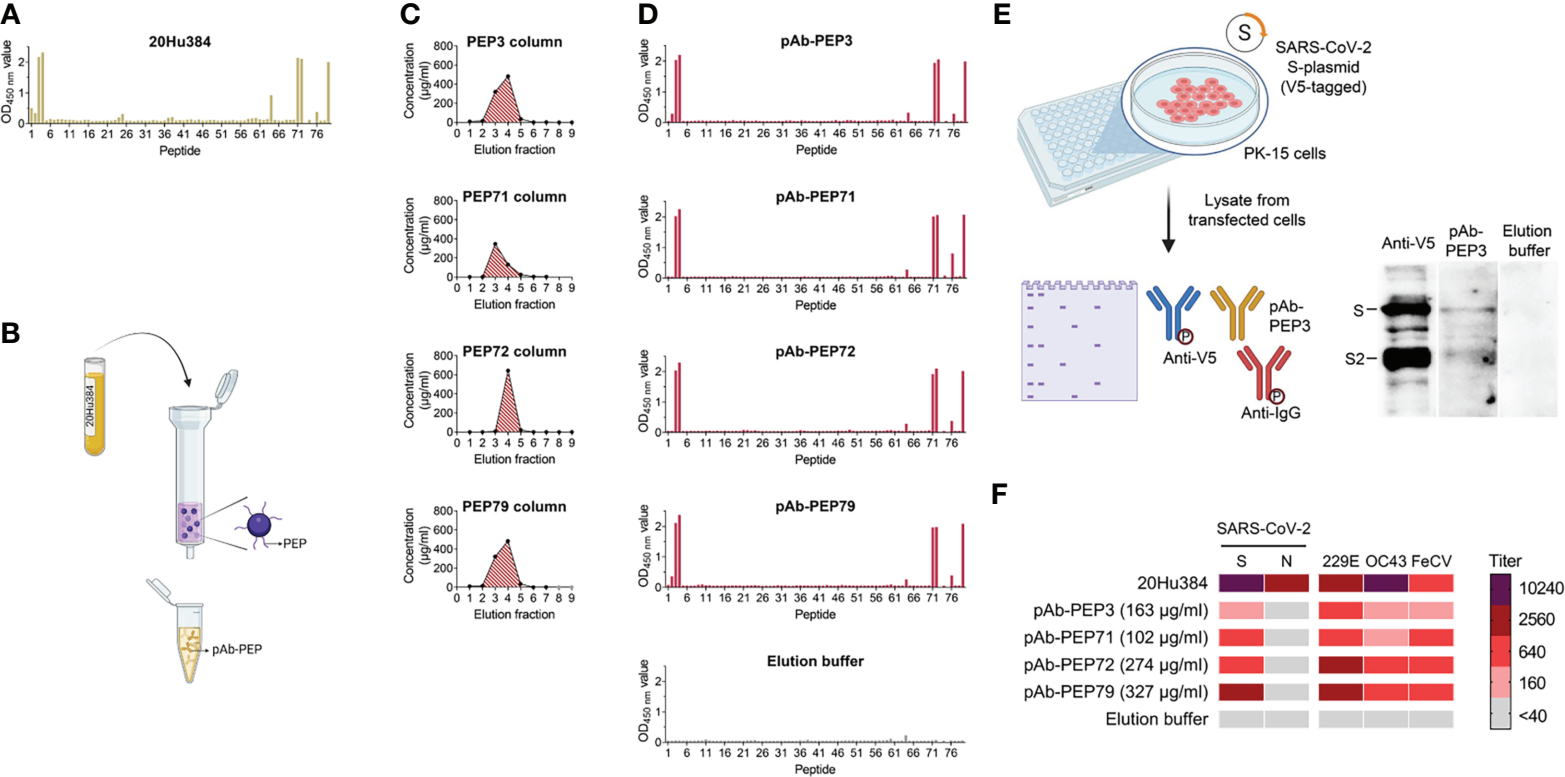
Isolation of peptide-purified antibodies (pAbs) from human serum. **(A)** Pepscan analysis of human serum sample #20Hu384 (obtained from a COVID-19 convalescent patient) prior to purification. **(B)** Purification principle. Serum #20Hu384 was submitted to peptide-affinity chromatography using immobilized PEP3, PEP71, PEP72 or PEP79. **(C)** Elution profiles during chromatography. The fractions with the highest concentrations were combined. **(D)** Pepscan analysis of the pAbs purified on columns with immobilized PEP3, PEP71, PEP72 or PEP79. The lowest panel shows the profile of the control sample containing late (low-protein) elution fractions (= fractions 8 and 9 from PEP79 column). The bars show the OD_450 nm_ value. **(E)** Western blot analysis of S-plasmid transfected PK-15 cells shows binding of pAb-PEP3 to cleaved (S2) and full-length SARS-CoV-2 S. **(F)** Antibody titers of serum #20Hu384, the four pAbs and elution buffer in IPMA assays for SARS-CoV-2 S and N; HCoV-229E and -OC43; and FeCV.

Knowing that PEP3-binding antibodies are commonly formed in COVID-19 patients and also present in some pre-pandemic samples, we wished to characterize their spike-binding and virus-neutralizing properties. To isolate these antibodies by peptide affinity chromatography (**Figure 9**), we used a serum available in relatively large amount from a COVID-19 convalescent individual (#20Hu384). After verifying its reactivity by pepscan (**Figure 9A**), four peptide-purified antibodies (pAbs) recognizing PEP3, PEP71, PEP72 and PEP79 were purified from this serum (**Figure 9B**). PEP79 contains the same sequence as PEP3, but with six extra amino acids (sequence in **Figure 5**). We added PEP79 because of the reported finding that depletion of pooled COVID-19 patient sera with this peptide (referred to as S21P2) reduced the SARS-CoV-2 neutralizing activity by about 20% (33). The peptides were covalently immobilized on an N-hydroxysuccinimide-activated sepharose column and bound serum antibodies were eluted as previously described (41). The elution fractions with the highest protein concentrations were combined from each column (**Figure 9C**). The pAbs were then assayed for reactivity and specificity, using four different techniques: pepscan (**Figure 9D**), western blot (**Figure 9E**), IPMA (**Figure 9F**) and surface plasmon resonance (SPR; **Supplemental Figure S4**). In pepscan analysis with our panel of 76 peptides, all four pAbs showed specific enrichment for antibodies against PEP3, -4, -71, -72, -76 and -79. pAb-PEP3 and pAb-PEP79 also weakly reacted to PEP2 (whose sequence also occurs in the longer PEP79 and N-terminally overlaps with PEP3). The pAbs lacked reactivity to PEP1, seen with serum #20Hu384 before it was purified (**Figure 9A**).

Denaturating PAGE on a SARS-CoV-2-S (V5-tagged)-positive cell lysate, followed by western blot detection with pAb-PEP3 and anti-V5 as the primary antibodies (**Figure 9E**), confirmed that pAb-PEP3 binds to a linear epitope that is present in the full-length spike and its S2 subunit. In addition, in IPMA assays, the four pAbs displayed titers of 160-2560 for SARS-CoV-2 S; 640-2560 for HCoV-229E; 160-640 for HCoV-OC43; and 160-640 for FeCV (**Figure 9F**). This corroborated the functionality and broad coronavirus reactivity of the PEP3-, PEP71-, PEP72- and PEP79-pAbs.

Using SPR (**Supplemental Figure S4**), we confirmed binding of PEP3, -71, -72 and -79 to the four corresponding pAbs. For each pAb, peptide PEP79 gave the highest RU signal, which is plausibly explained by its longer length (**Supplemental Figure S4A**). None of the peptides interacted with a commercial monoclonal antibody targeting the SARS-CoV-2 S2 subunit. Besides, we analyzed binding of the pAbs to full-length SARS-CoV-2 S protein or its subdomains (**Supplemental Figure S4B**). Alike the commercial anti-S2 antibody, all four pAbs bound to full-length S protein and its S2 subunit, while they did not interact with the receptor binding domain (RBD) or S1 subunit. In contrast, the RBD-targeting REGN10987 antibody [imdevimab (53)] did not bind to S2 while exhibiting clear binding to the RBD, S1 and full-length S protein.

Finally, we determined the reactivity of pAb-PEP79 against a series of additional peptides (PEP80-89) derived from the free fusion peptide (i.e. lacking the S2′ cleavage site) of HCoV-OC43 and HCoV-229E; MERS-CoV sequence (with or without the S2′ site); and PEP79 homologs of MERS-CoV, SARS-CoV, FeCV and FIPV (serotypes I and II). Besides reacting with PEP3, -4, -71 and -72, pAb-PEP79 reacted strongly with the entire PEP80-89 series, regardless of a few amino acid variations in the FP sequence. This firmly qualifies the pAbs as broad-coronavirus spike-binding antibodies (**Supplemental Figure S5**).

### Despite Broad Spike-Binding Capacity, the pAbs Exhibit Poor Virus-Neutralizing Activity

**Figure 10:**
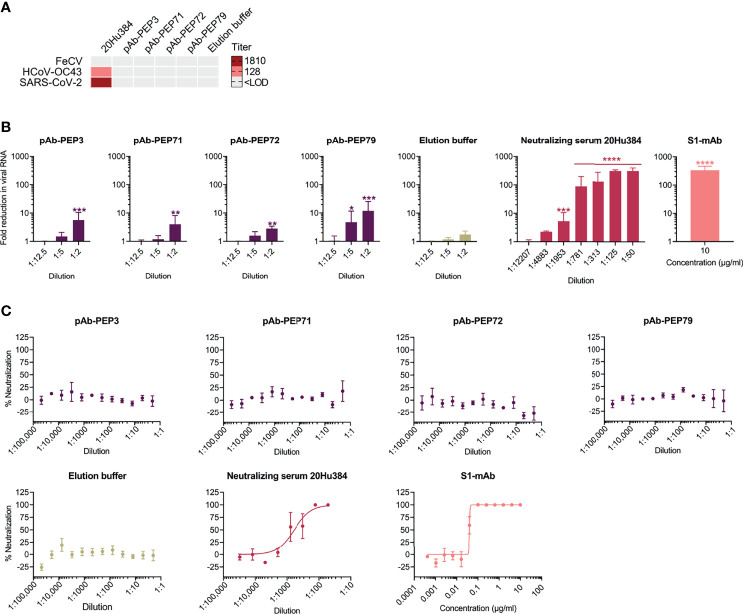
Virus-neutralizing properties of peptide-purified human antibodies and parent serum 20Hu384. **(A)** The virus-neutralizing activity was determined by seroneutralization (SN) assay against FeCV in feline enterocytes, HCoV-OC43 in HRT-18 cells, and SARS-CoV-2 in VeroE6 cells. The limit of detection (LOD) was a 1:8 (FeCV) or 1:40 dilution (HCoV-OC43 and SARS-CoV-2). **(B, C)** Evaluation of the pAbs (starting dilution: 1:2) in a SARS-CoV-2 neutralization assay in Calu-3 cells. **(B)** Evaluation based on viral load quantification at 24 h p.i. The three right panels show the negative control (elution buffer); parent human serum #20Hu384; and positive control antibody (S1-mAb: SARS-CoV-2 neutralizing anti-S1 antibody). Data are the mean ± SEM of two independent experiments, performed in duplicate. The Y-axis shows the fold reduction in viral RNA versus untreated virus control, based on qRT-PCR analysis of the supernatants at 24 h p.i. *P ≤ 0.05; **P ≤ 0.01; ***P ≤ 0.001, ****P ≤ 0.0001 (multiple unpaired t-test with Holm-Šídák correction; treated sample versus untreated virus control). **(C)** In parallel, virus replication was quantified by immunofluorescence staining for dsRNA at 72 h p.i. The graphs represent the percentage neutralization in function of antibody dilution or concentration.

Lastly, we evaluated the four pAbs in virus neutralization assays (**Figure 10**). Neither of them showed neutralizing activity for FeCV in feline enterocytes (**Figure 10A**), as also seen with the parent human serum (#20Hu384) the pAbs were concentrated from. Likewise, there was no neutralization of HCoV-OC43 in HRT-18 cells and of SARS-CoV-2 in VeroE6 cells (**Figure 10A**), while the parent human serum did neutralize both viruses (SN titers of 128 against HCoV-OC43 and 1810 against SARS-CoV-2).

In VeroE6 cells, SARS-CoV-2 enters *via* the endosomal pathway in which S2′ cleavage is carried out by endo/lysosomal cathepsins. In contrast, in human airway cells, the spike is predominantly cleaved by airway proteases such as TMPRSS2 (54). To exclude the possibility that the lack of virus neutralization observed with the pAbs was due to the virus entry route, we also conducted a neutralization experiment in the airway epithelium Calu-3 cell model (**Figures 10B, C**). When virus replication was measured at 24 h p.i., using RT-qPCR for viral RNA in the supernatants, low (3- to 12-fold) though significant (P<0.01 *versus* untreated virus control) reductions in viral RNA were seen at the lowest dilution (1:2) of the four pAbs (**Figure 10B**). pAB-PEP79 was slightly more active, giving still 5-fold reduction (P=0.016) at 1:5 pAb dilution. However, when replication was measured at 72 h p.i., using immunostaining, the pAbs proved inactive whereas the parent serum (#20Hu384) and a commercial neutralizing anti-S1-antibody did neutralize the virus (**Figure 10C**). The purified pAbs thus exhibited dose-dependent yet weak neutralizing potency, causing only transient reduction of SARS-CoV-2 infection in Calu-3 cells.

In short, we conclude that antibodies targeting the N-terminal part of the SARS-CoV-2 FP exhibit cross-reactive binding towards the spikes of human and animal alpha- or betacoronaviruses, providing weak inhibition of SARS-CoV-2 entry into the host cells.

## Discussion

The spike proteins of coronaviruses elicit high titers of virus- or variant-specific antibodies, many of which bind to the receptor-binding S1 subunit (55, 56). Still, identification of more conserved epitopes, particularly in the S2 subunit (57), will help to develop cross-reactive therapeutic antibodies that cover several coronaviruses and variants. As evident from our sensitive IPMA analyses on pre-pandemic samples and on sera from a longitudinal COVID-19 patient cohort, cross-reactive antibodies are commonly formed during coronavirus infection. Consistent with HCoV-OC43 and HCoV-229E being widespread causes of upper respiratory infections, we found that antibodies against these viruses were present in almost all our pre-pandemic samples and in all our COVID-19 patients at symptom onset. Two earlier studies, both based on ELISA, also found near-universal seropositivity for endemic CoVs in adults (58, 59). Our HCoV-229E and -OC43 IPMA assays broadly detect antibodies against all viral proteins expressed in infected cells. Since these proteins can contain CoV cross-reactive epitopes, our data do not allow to estimate the seroprevalence rates for HCoV-229E and -OC43 specifically. As for the reactivity of our pre-pandemic samples towards SARS-CoV-2 S, 2.2% of the samples were overtly positive and 48% showed limited cross-reactivity (titer 40-160). A low rate (2-5%) of cross-reactivity was reported by others (17, 27) while one study detected up to 30% cross-reactivity, albeit at low levels (26). Finally, the limited cross-reactivity (24%) of our pre-pandemic plasma samples towards SARS-CoV-2 N aligns with another study (27). Reciprocally, the COVID-19 patients in our study showed a significant rise in HCoV-OC43 but not HCoV-229E cross-reacting antibodies, as also seen by others (26, 27). We observed a temporal trend in OC43 titers with progressing SARS-CoV-2 infection, monitored by measuring neutralizing antibody titers, and N- and S-IPMA titers. A concomitant increase in these four antibody titers was observed starting the second week post-symptom onset and these antibodies remained elevated during the four weeks of our study. Whether the antibodies elicited by SARS-CoV-2 are cross-reactive to conserved betacoronavirus antigens or originate from a recall of pre-existing anti-HCoV-OC43 memory B cells is unclear (47). Still, at the levels induced by natural infection, these broad antibodies may afford little protective effect. Indeed, Anderson et al. (27) found no correlation between the occurrence of pre-pandemic HCoV-OC43 or SARS-CoV-2 cross-reactive antibodies and protection against SARS-CoV-2 infection or severe COVID-19.

To identify linear epitopes that are able to elicit cross-reactive anti-spike antibodies, we focused on a region that is highly conserved among human alpha- and betacoronaviruses; spans residues 806-1091 of SARS-CoV-2 S2; and corresponds to the S2′ cleavage site, FP and heptad repeat 1. Combining our data with those of others (14–16, 19, 33–35), we can conclude that this S2 region exhibits notably high and consistent immunogenicity. More specifically, in pepscan analysis, all our COVID-19 patient sera and some pre-pandemic samples showed high reactivity for the PEP3 region (814-KRSFIEDLLFNK-825), which encompasses the S2′ cleavage site and N-terminal fragment of the FP. A similarly high PEP3 reactivity was seen in some sera belonging to either cohort, suggesting that PEP3 binding was comparably efficient whether the antibodies were elicited by the cognate (i.e. SARS-CoV-2) S2 sequence or an endemic HCoV homologue. Our observation that also sera from FeCV-infected cats exhibit PEP3 reactivity suggests that this short sequence in S2 is widely immunogenic for coronaviruses. Based on this, we selected PEP3, its homologs from HCoV-OC43 and HCoV-229E, plus the longer peptide PEP79, to isolate peptide-purified antibodies. This yielded four pAbs with SPR-confirmed binding activity towards the corresponding peptides, full-length SARS-CoV-2 S protein and its S2 subunit. Their pan-coronavirus spike-binding capacity was demonstrated in IPMA assays with diverse alpha- and betacoronaviruses and in pepscan assays with PEP3-homologous peptides derived from SARS-CoV-2, HCoV-OC43, HCoV-229E, MERS-CoV, SARS-CoV, FeCV and FIPV. Nevertheless, a limitation of this antibody purification technique is its inability to discriminate polyclonal from monoclonal antibodies.

To conclude, besides being readily formed during a natural coronavirus infection, antibodies targeting linear epitopes around the PEP3 region seem quite effective at binding the S protein.

On the other hand, the four pAbs were shown to minimally inhibit SARS-CoV-2 infection and lack HCoV-OC43 or FeCV neutralizing activity. This is in line with other studies in which different antibody preparations, peptides or methodologies than ours were used. A peptide identical to PEP79 (S21P2; PSKPSKRSFIEDLLFKV) only modestly reduced the SARS-CoV-2 S-pseudovirus neutralizing effect, when pooled COVID-19 patient sera were depleted with this peptide (33). Convalescent human sera purified with peptide PSKRSFIEDLLF proved non-neutralizing towards SARS-CoV-2 S-pseudovirus at 21 µg/ml (32). In our study, the lowest antibody concentration giving a significant SARS-CoV-2 RNA reduction was in the range of 50-65 µg/ml (pAb-PEP71 and pAb-PEP79 at 1:2 and 1:5 dilution, respectively). The marginal neutralizing effect was also seen in animal studies. Sera from mice immunized with peptide PSKRSFIEDLLF or peptide IEDLLFNKVTLA were unable to neutralize SARS-CoV-2 (60). Also, immunization of pigs with a 24-mer peptide spanning the S2′ cleavage site of a porcine alphacoronavirus (porcine epidemic diarrhea virus, PEDV) or with the homologous peptide from SARS-CoV-2, did not elicit PEDV-neutralizing antibodies before PEDV challenge (61). Finally, sera from rabbits immunized with a long peptide encompassing the fusion peptide (S7,972–8,013) showed moderate neutralization activity against SARS-CoV-2 pseudotypes in Calu-3 cells (62).

What could be the reason for this weak virus neutralization despite clear evidence, in IPMA assays, that our pAbs effectively bind to the spike proteins of different coronaviruses? It is plausible that the S2′ and FP sequence targeted by these pAbs is only briefly exposed during the short process of virus fusion with the cell membrane. When coronaviruses bind to cell surface receptors, the S2′ site is cleaved by nearby membrane proteases (20, 25, 63) to release the FP and induce membrane fusion. In theory, the pAbs might be able to prevent virus entry by sterically hindering the S2′ cleavage or by binding the FP and preventing its membrane insertion (19). However, the weak SARS-CoV-2 neutralizing effect in Calu-3 cells (which are rich in relevant proteases like TMPRSS2) suggests that our pAbs had difficulties in accessing their epitope. Some technical issue is very unlikely, given the strong efficacy of small molecule protease inhibitors like camostat in our SARS-CoV-2/Calu-3 cell model (54). Instead, it is possible that the epitope of the pAbs might be inaccessible until ACE2 binding triggers a subtle conformational change in the S2′ loop, required for cleavage and FP exposure (64, 65). The structural change might possibly enable rapid S2′ cleavage in most protomers, before the pAbs can bind to this region and block the fusion process. The use of nanobodies targeting this region could be an interesting option to enhance epitope accessibility. Alternatively, since our pAbs target the N-terminal helix of the FP, they may be ineffective at inhibiting membrane penetration by its second amphipathic helix (66).

Our findings point to the FP region as being potentially relevant for designing antibodies or immunogens that cover multiple coronaviruses or variants. Interestingly, the FPs of other viral class I fusion proteins were already recognized as a common site of vulnerability. Broadly neutralizing antibodies (bnAbs) interacting with the FP were identified for influenza virus, Ebola virus and HIV (67).

*In vivo*, non-neutralizing antibodies can still have Fc-mediated effector functions related to complement-dependent cytotoxicity, antibody-dependent cell-mediated cytotoxicity (ADCC) or opsonization for phagocytosis (68). Non-neutralizing antibodies targeting the SARS-CoV-2 spike were shown to induce ADCC (69). Also, an early rise in S2-specific Fc-receptor binding antibodies was observed in individuals who survived severe COVID-19 (70). It is possible that anti-FP antibodies might carry such Fc-mediated functions. In the context of SARS-CoV-2 vaccination, they might complement anti-S1 antibodies to provide not only first line (virus neutralization) but also second line defense (recognition of infected cells), offering protection against severe COVID-19 (70, 71).

In conclusion, cross-reactive antibodies are commonly elicited during coronavirus infection, with the N-terminal region of the FP being widely immunogenic in this setting.

## Data Availability Statement

The raw data supporting the conclusions of this article will be made available by the authors, without undue reservation.

## Ethics Statement

The studies involving human participants were reviewed and approved by UZ-Ghent ethics committee approval is BC-07829. The patients/participants provided their written informed consent to participate in this study. Ethical review and approval were not required for the animal study because only leftover samples that were submitted for diagnostic purposes to the Laboratory of Virology of the Faculty of Veterinary Medicine, Ghent University, were used. All samples were pseudonymised.

## Author Contributions

HN, JX, NVa, AS, and LN designed research. JX, NVa, AS, XR, CB, and SN performed research. NVe and DO analyzed data. ID, BV, PG, VS, and MV contributed materials and NVa, AS, and LN wrote the paper. All authors contributed to the article and approved the submitted version.

## Funding

JX was funded by a BOF project at Ghent University. NVe is supported by a grant from the Flemish Agency for Innovation and Entrepreneurship (Baekeland Mandate HBC.2020.2889). VS is funded by a PhD fellowship from the Research Foundation Flanders (application number 1192120N). LN acknowledges funding from the European Union’s Innovative Medicines Initiative (IMI) under Grant Agreement 101005077 [Corona Accelerated R&D in Europe (CARE) project] and Fundació La Marató de TV3, Spain (Project No. 201832-30).

## Conflict of Interest

The authors declare that the research was conducted in the absence of any commercial or financial relationships that could be construed as a potential conflict of interest.

## Publisher’s Note

All claims expressed in this article are solely those of the authors and do not necessarily represent those of their affiliated organizations, or those of the publisher, the editors and the reviewers. Any product that may be evaluated in this article, or claim that may be made by its manufacturer, is not guaranteed or endorsed by the publisher.
