# Supplementary material for: Functional Analysis of Human and Feline Coronavirus Cross-Reactive Antibodies Directed Against the SARS-CoV-2 Fusion Peptide
Source: Front Immunol. 2022 Jan 5;12:790415. doi: 10.3389/fimmu.2021.790415 (PMC8766817; doi:10.3389/fimmu.2021.790415)
Supplement: Supplementary file 1 [file DataSheet_1.pdf]

Supplementary Material

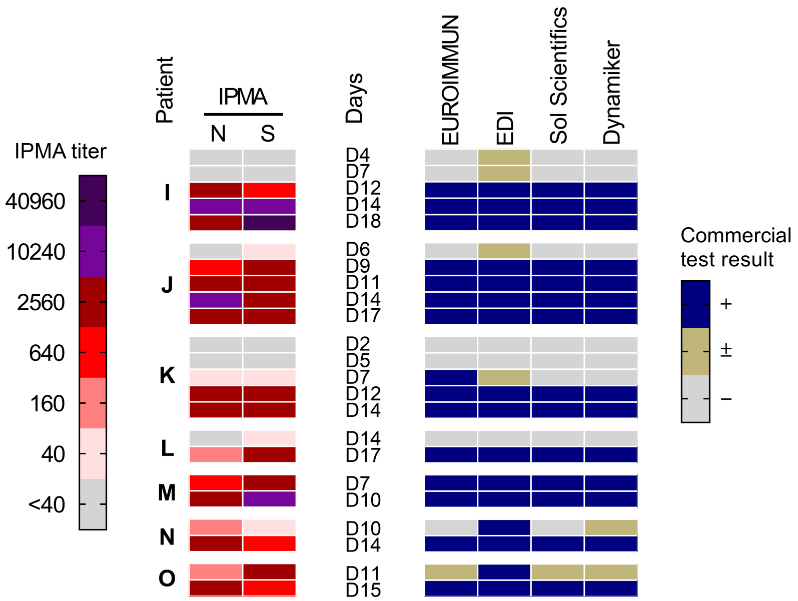

Supplementary Figure 1. Performance of the SARS-CoV-2 N- and S-IPMA assays in comparison to four commercial tests for detection of SARS-CoV-2 antibodies in serum. In total, 23 sera from 7 COVID-19 patients were analysed. The data shown on the left (IPMA assays) are the same as in Figure 2. On the right are the test results from commercial SARS-CoV-2 serological assays from EUROIMMUN (anti-SARS-CoV-2 ELISA IgG); Epitope Diagnostics (EDI, Novel Coronavirus COVID-19 IgG ELISA Kit); Sol Scientifics (Coronavirus Disease Combined IgM/IgG Rapid Test); and Dynamiker Biotechnology (2019 nCOV IgG/IgM Rapid Test). Symbols: (-) negative, (+) positive and (+/-) borderline, according to the serological test manufacturer.

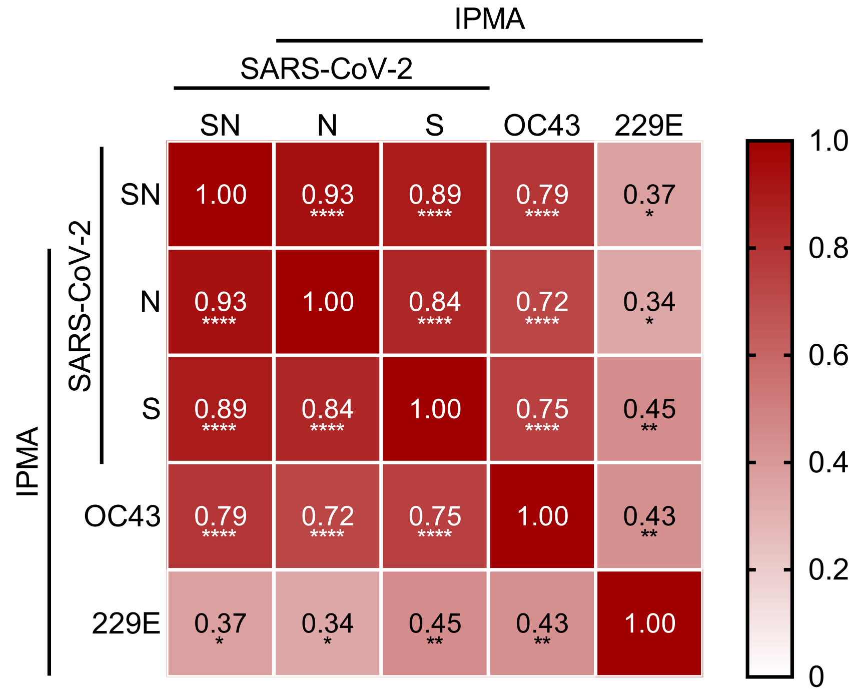

Supplementary Figure 2. Pearson correlation between SARS-CoV-2 neutralizing antibody titer and IPMA-based antibody titers against SARS-CoV-2 N and S, HCoV-OC43 and HCoV-229E. The titers used for correlation analysis were from COVID-19 patients A-O (see Figure 3 in the main text). Inside the correlation matrix, the Pearson correlation efficiency (r) and p-value (two-tailed) are provided for each pairwise analysis.

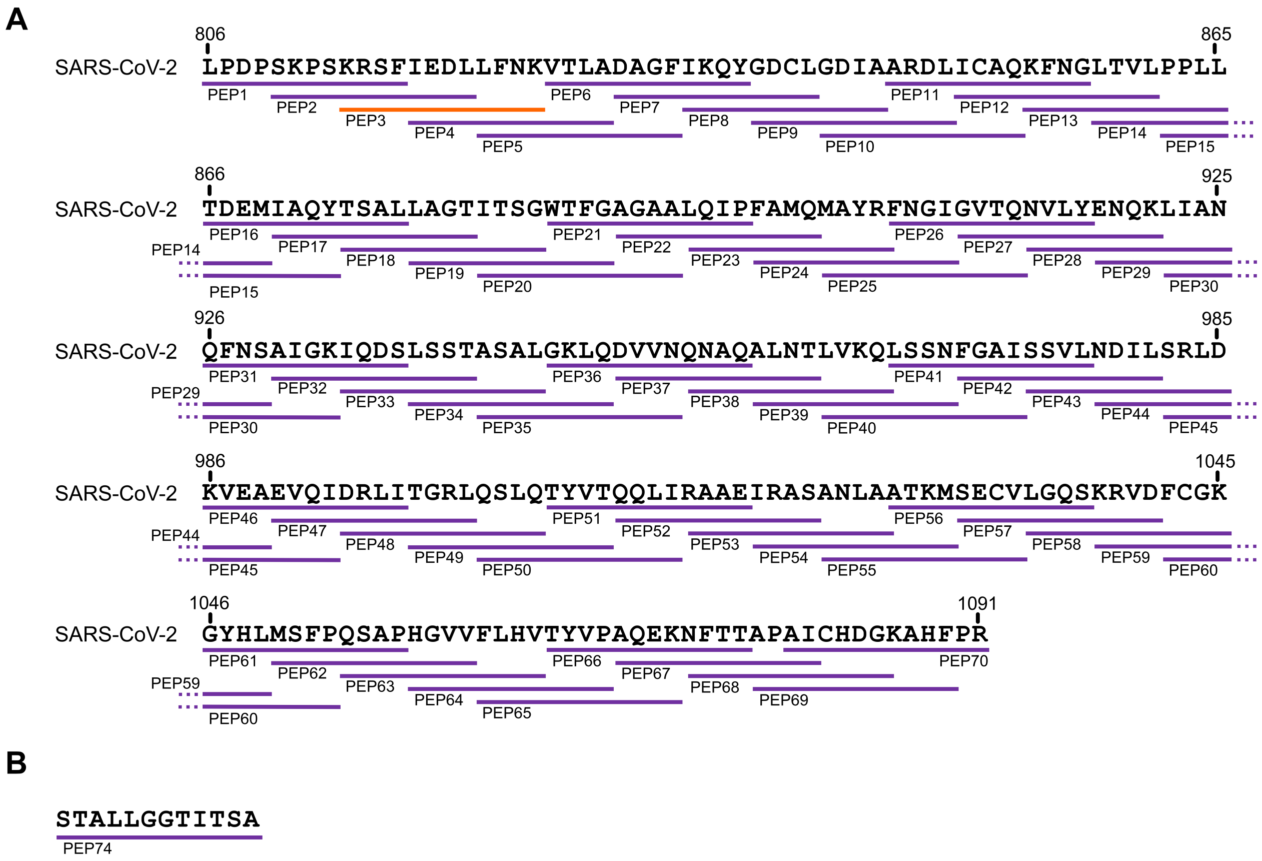

Supplementary Figure 3. Visualization of amino acid sequences of SARS-CoV-2 peptides 1-70. (A) Partial spike sequence from SARS-CoV-2 (NCBI accession number: YP_009724390) with the PEP sequences in purple bars. (B) Sequence of the control peptide PEP74 (= scrambled sequence of PEP18).

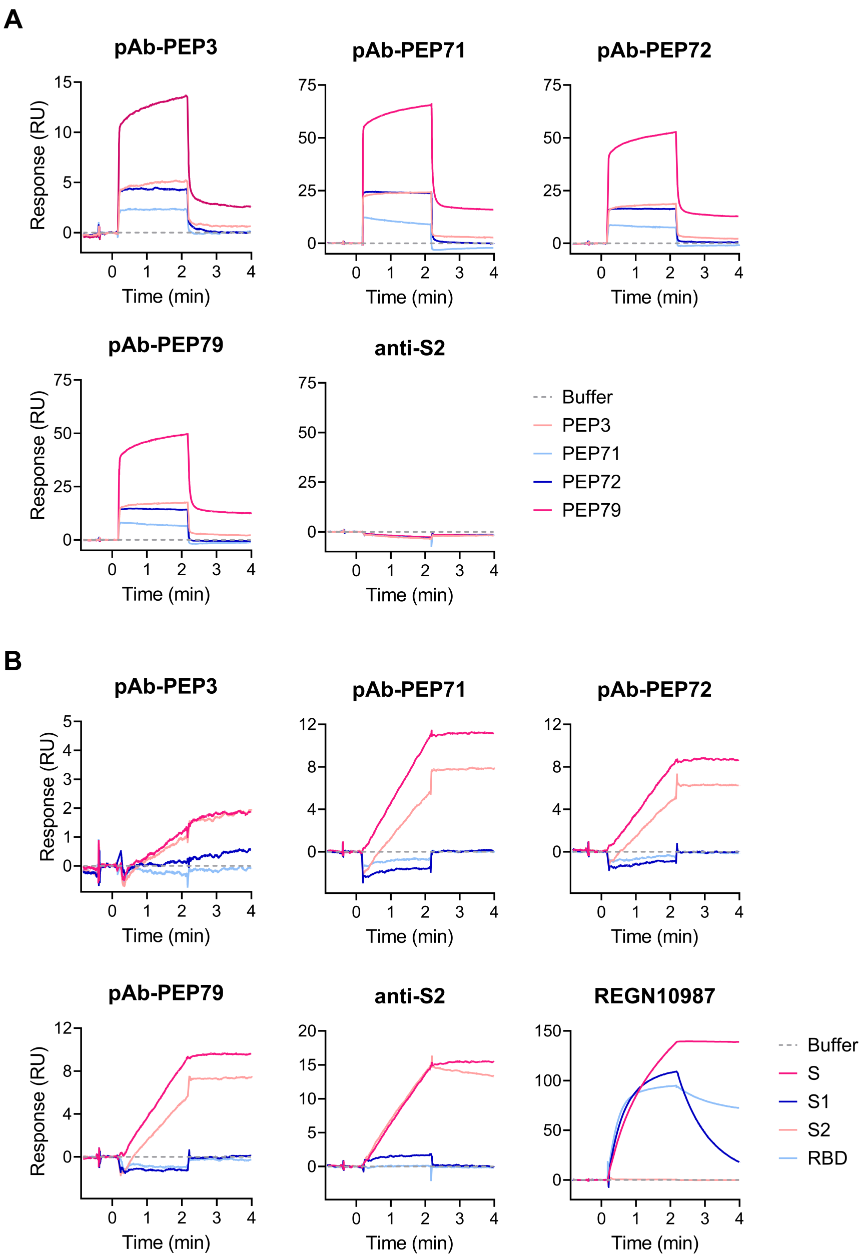

Supplementary Figure 4. SPR analysis confirmed that the pAbs bind the S2'-FP peptides as well as the SARS-CoV-2 spike protein and its S2 subunit. Sensorgrams represent binding to one of the four pAbs or control antibodies, which were captured by anti-human IgG on a CM5 chip. The Y-axis shows the resonance signal in resonance units (RU), while the X-axis represents the duration of the measurement (in minutes). For both axes, 0 represents the baseline. (A) Binding of PEP3, -71, -72 and -79 (all diluted 1:100) to pAb-PEP3 (420 RU captured); pAb-PEP71 (2024 RU captured); pAb-PEP72 (1695 RU captured); pAb-PEP79 (1660 RU captured); and commercial anti-S2 control antibody (535 RU captured). (B) Binding of full-length SARS-CoV-2 spike protein (S; 20 nM), or its S2, S1 or receptor binding domains (all at 50 nM) to pAb-PEP3 (406 RU captured); pAb-PEP71 (2020 RU captured); pAb-PEP72 (1814 RU captured); and pAb-PEP79 (1884 RU captured). Commercial anti-S2 Ab (432 RU captured) and REGN10987 (247 RU captured) were included as controls. Note that the Y-axes are different for some antibodies.

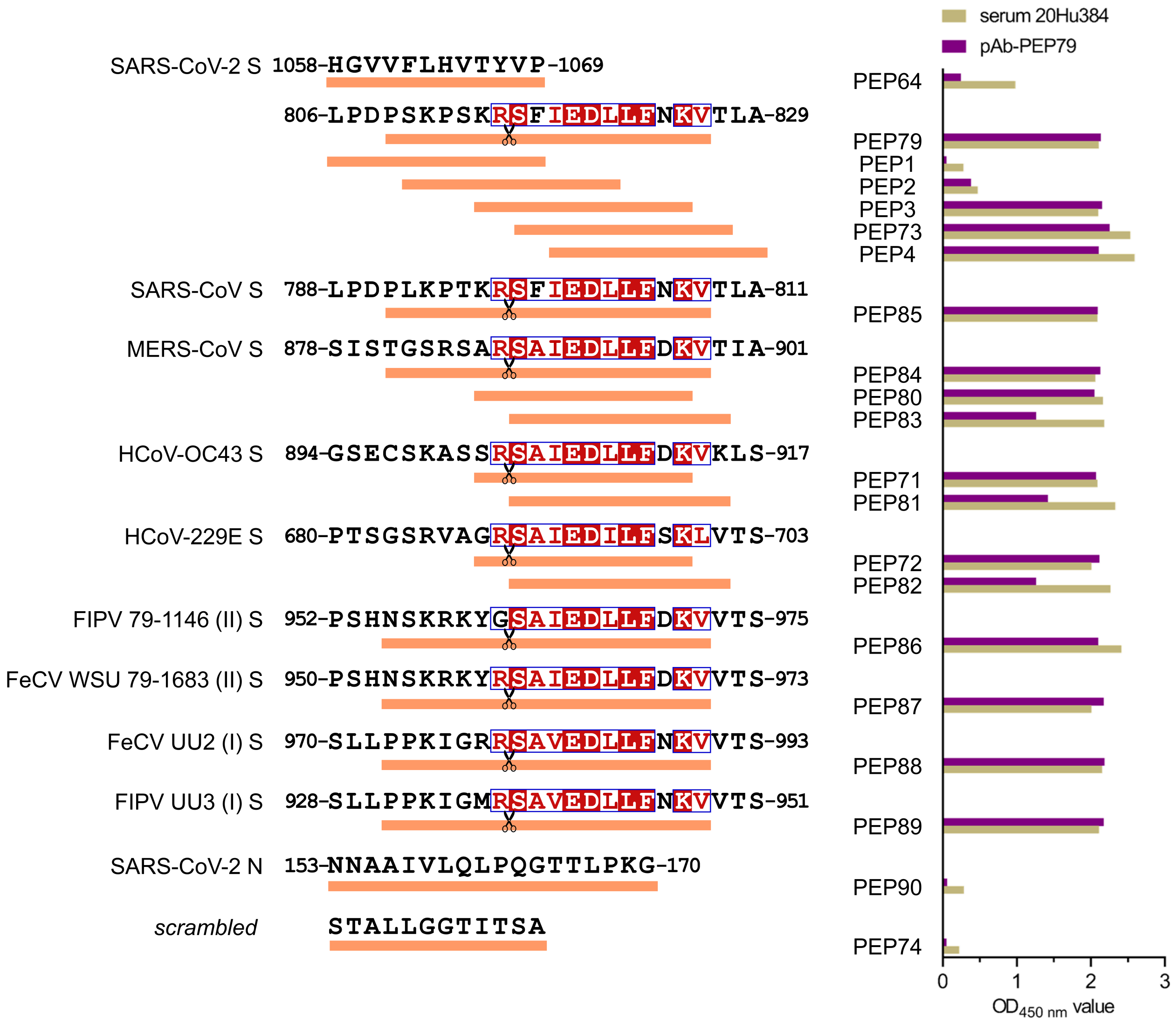

Supplementary Figure 5. Additional peptides indicate pan-coronavirus reactivity of pAb-PEP79. The sequence of each peptide is indicated by a coloured bar spanning the amino acid sequence from which it is derived (left). The S2' cleavage site is indicated by the scissors. The reactivity of pAb-PEP79 and its parent serum 20Hu384 in a pepscan with these immobilized peptides is shown in the bar graphs (absolute $OD_{450\ nm}$ value) on the right. Sequence similarities from Clustal Omega-aligned sequences were rendered using ESPript 3.0. Shown in red shading: fully conserved residues; in red font: residues are similar according to physicochemical properties; and boxed in blue: ≥70% similarity. NCBI accession numbers: YP_009724390 (SARS-CoV-2 S), YP_009825051 (SARS-CoV S), YP_009047204 (MERS-CoV S), YP_009555241 (HCoV-OC43 S), NP_073551 (HCoV-229E S), YP_004070194 (FIPV 79-1146 S), AFH58021 (FeCV WSU 79-1683 S), ACT10948 (FeCV UU2 S), ACT10959 (FIPV UU3 S) and YP_009724397 [SARS-CoV-2 N; peptide N4P5 (Amrun et al., 2020)].
